# Bacterial Outer Membrane Polysaccharide Export (OPX) Proteins Occupy Three Structural Classes with Selective β-Barrel Porin Requirements for Polymer Secretion

**DOI:** 10.1128/spectrum.01290-22

**Published:** 2022-10-06

**Authors:** Fares Saïdi, Utkarsha Mahanta, Adyasha Panda, Ahmad A. Kezzo, Nicolas Y. Jolivet, Razieh Bitazar, Gavin John, Matthew Martinez, Abdelkader Mellouk, Charles Calmettes, Yi-Wei Chang, Gaurav Sharma, Salim T. Islam

**Affiliations:** a Institut National de la Recherche Scientifique (INRS), Centre Armand-Frappier Santé Biotechnologie, Université du Québec, Institut Pasteur International Network, Laval, Quebec, Canada; b PROTEO, the Quebec Network for Research on Protein Function, Engineering, and Applications, Université Laval, Québec, Quebec, Canada; c Institute of Bioinformatics and Applied Biotechnology (IBAB), Bengaluru, Karnataka, India; d Department of Pediatrics, Division of Infectious Diseases, Children’s Hospital of Philadelphia, Philadelphia, Pennsylvania, USA; e Department of Biochemistry and Biophysics, Perelman School of Medicine, University of Pennsylvania, Philadelphia, Pennsylvania, USA; Centre National de la Recherche Scientifique, Aix-Marseille Université

**Keywords:** Wzx/Wzy-dependent pathway, biofilms, capsule polysaccharide, ABC transporter-dependent pathway, genomics, outer membrane, outer membrane proteins, DUF6029, periplasm thickness, synthase-dependent pathway, porins, secretion systems

## Abstract

Secretion of high-molecular-weight polysaccharides across the bacterial envelope is ubiquitous, as it enhances prokaryotic survival in (a)biotic settings. Such polymers are often assembled by Wzx/Wzy- or ABC transporter-dependent schemes implicating outer membrane (OM) polysaccharide export (OPX) proteins in cell-surface polymer translocation. In the social predatory bacterium Myxococcus xanthus, the exopolysaccharide (EPS) pathway WzaX, major spore coat (MASC) pathway WzaS, and biosurfactant polysaccharide (BPS) pathway WzaB were herein found to be truncated OPX homologues of Escherichia coli Wza lacking OM-spanning α-helices. Comparative genomics across all bacteria (>91,000 OPX proteins identified and analyzed), complemented with cryo-electron tomography cell-envelope analyses, revealed such “truncated” WzaX/S/B architecture to be the most common among three defined OPX-protein structural classes independent of periplasm thickness. Fold recognition and deep learning revealed the conserved M. xanthus proteins MXAN_7418/3226/1916 (encoded beside *wzaX/S/B*, respectively) to be integral OM β-barrels, with structural homology to the poly-*N*-acetyl-d-glucosamine synthase-dependent pathway porin PgaA. Such bacterial porins were identified near numerous genes for all three OPX protein classes. Interior MXAN_7418/3226/1916 β-barrel electrostatics were found to match properties of their associated polymers. With MXAN_3226 essential for MASC export, and MXAN_7418 herein shown to mediate EPS translocation, we have designated this new secretion machinery component “Wzp” (i.e., Wz porin), with the final step of M. xanthus EPS/MASC/BPS secretion across the OM now proposed to be mediated by WzpX/S/B (i.e., MXAN_7418/3226/1916). Importantly, these data support a novel and widespread secretion paradigm for polysaccharide biosynthesis pathways in which those containing OPX components that cannot span the OM instead utilize β-barrel porins to mediate polysaccharide transport across the OM.

**IMPORTANCE** Diverse bacteria assemble and secrete polysaccharides that alter their physiologies through modulation of motility, biofilm formation, and host immune system evasion. Most such pathways require outer membrane (OM) polysaccharide export (OPX) proteins for sugar-polymer transport to the cell surface. In the prototypic Escherichia coli Group-1-capsule biosynthesis system, eight copies of this canonical OPX protein cross the OM with an α-helix, forming a polysaccharide-export pore. Herein, we instead reveal that most OPX proteins across all bacteria lack this α-helix, raising questions as to the manner by which most secreted polysaccharides actually exit cells. In the model developmental bacterium Myxococcus xanthus, we show this process to depend on OPX-coupled OM-spanning β-barrel porins, with similar porins encoded near numerous OPX genes in diverse bacteria. Knowledge of the terminal polysaccharide secretion step will enable development of antimicrobial compounds targeted to blocking polymer export from outside the cell, thus bypassing any requirements for antimicrobial compound uptake by the cell.

## INTRODUCTION

Diverse bacteria associated with biotic and abiotic settings secrete high-molecular-weight polysaccharides across the cell envelope to enhance their survival. Capsule polysaccharide (CPS) chains are tightly bound to the cell surface and form hydrated exclusionary barriers to molecule entry, whereas exopolysaccharide (EPS) polymers form a more loosely surface-associated glycocalyx (1). Polysaccharides can also be secreted to the extracellular milieu, where they can influence bacterial physiology (2–4), with multiple secreted polymers often acting in concert to modulate complex physiology (5–7).

Myxococcus xanthus is a social and predatory Gram-negative motile soil bacterium (8–10). Under nutrient deprivation, cells in a swarm biofilm form myxospore-filled fruiting bodies through a developmental program resulting in a differentiated cell community (8). This intricate multicellular lifestyle is modulated by the secretion of three polysaccharides across the outer membrane (OM) (5). Cells constitutively produce a glycocalyx of EPS (2) which slows cell growth (11) and constitutes the main matrix component in M. xanthus biofilms (12, 13). A biosurfactant polysaccharide (BPS) is also synthesized but is instead secreted to the extracellular milieu (3), where it functionally destabilizes the EPS glycocalyx, leading to a range of fundamental behavioral and surface-property changes at the single-cell level (2). Spatiospecific production and synergy between EPS and BPS (3) impact M. xanthus swarm biofilm internal architecture, as well as their type IV pilus (T4P)-dependent expansion due to impacts on T4P production, stability, and positioning (2). Finally, the major spore coat (MASC) polymer is produced by spore-forming cells to protect nascent myxospores from environmental stresses (14, 15).

**FIG 1:**
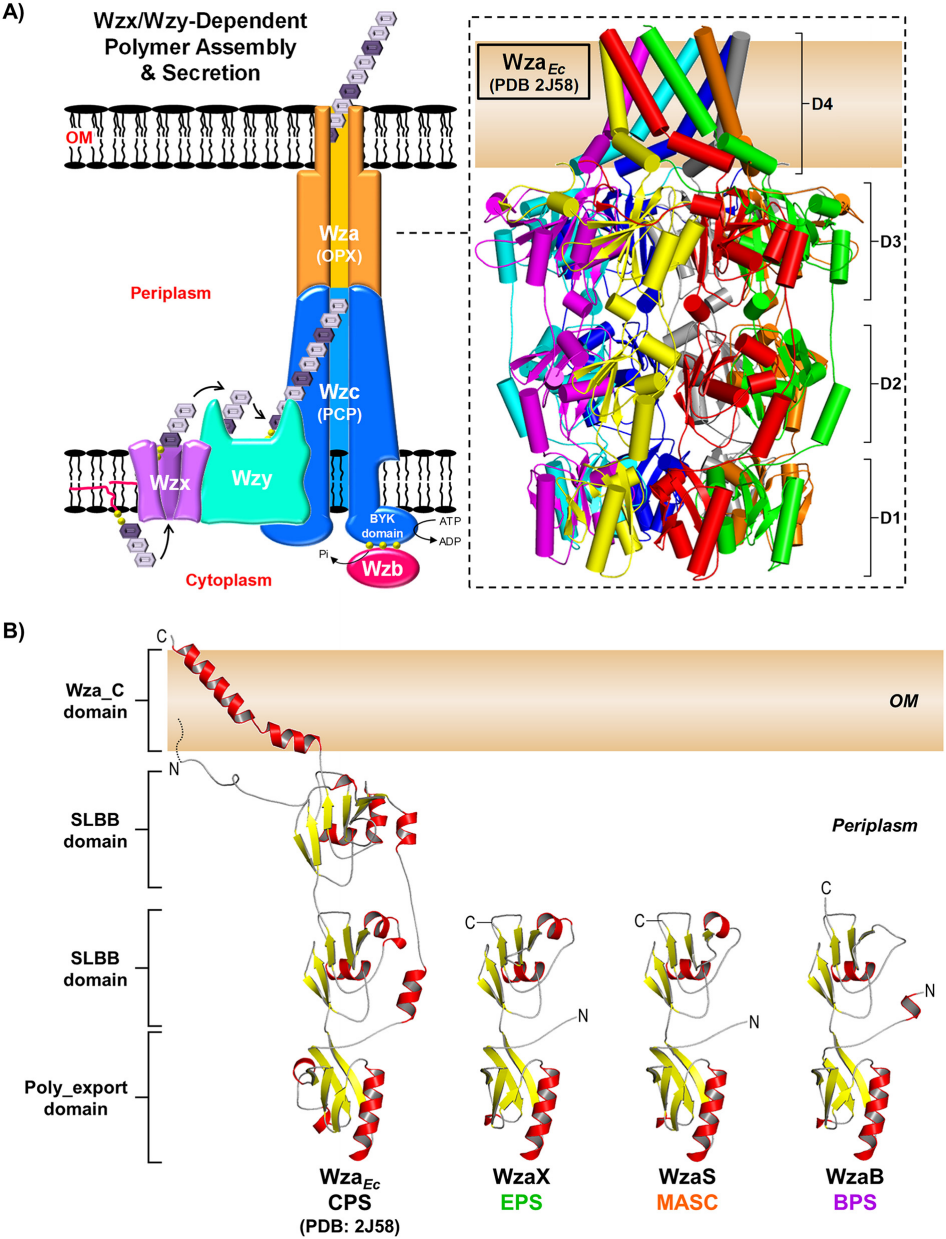
Conventional Wzx/Wzy-dependent polysaccharide assembly and secretion scheme. (A) Pathway schematic. (Inset) The Wza*_Ec_* X-ray crystal structure octamer (PDB: 2J58) is differentially colored to highlight the position of each chain in the structure. The Poly_export, SLBB, and Wza_C domains are indicated with smooth loops. (B) Tertiary structure models of M. xanthus EPS-pathway WzaX (aa 51 to 212), MASC-pathway WzaS (aa 32 to 190), and BPS-pathway WzaB (aa 38 to 202) based on the Wza*_Ec_* structure (aa 22 to 376, depicted with an N-terminal lipid anchor). Structures are displayed with smooth loops, highlighted β-sheets (yellow), and α-helices (red), with the various N and C termini indicated.

Each of these M. xanthus polysaccharides is produced by a separate Wzx/Wzy-dependent pathway (3, 14, 16, 17), the components for which have the suffixes X (exopolysaccharide), B (biosurfactant), or S (spore coat). Therein, Wzx flippases translocate undecaprenyl pyrophosphate (UndPP)-linked sugar repeats across the inner membrane (IM) (18–20), followed by polymerization at the periplasmic leaflet by Wzy (21, 22), to lengths governed by the polysaccharide co-polymerase (PCP) Wzc (Fig. 1A) containing either an integrated or associated (Wze) tyrosine autokinase (1, 3, 23). In turn, the Wzb tyrosine phosphatase regulates the state of PCP-associated phosphorylation (24). Tyrosine kinase dephosphorylation via Wzb has been proposed to drive Wzc octamerization, in turn affecting Wzy-mediated polymerization and interaction with the outer-membrane polysaccharide export (OPX) Wza protein needed for polymer translocation through the periplasm and across the OM (25) (Fig. 1A). Such bacterial pathways are widespread, generating diverse products such as Group 1 and Group 4 (i.e., O antigen) CPS, as well as colanic acid polymers in enterobacteria (26), in addition to holdfast (27) and xanthan (28). In Group 1 CPS systems, the 18-stranded integral OM β-barrel Wzi (internally occluded by an α-helical plug domain) is also important, as it displays lectin-like characteristics implicated in capsule structure organization (29).

Secreted polymers can also be synthesized by an ABC transporter-dependent scheme in which UndPP-linked polymers are built by sugar unit addition at the cytoplasmic leaflet of the IM, with the polymer generated entirely in the cytoplasm. Subsequent ATP hydrolysis by the transporter drives polymer transport across the IM, after which PCP and OPX proteins are needed for the secretion of polymer through the periplasm and across the OM. These biosynthesis pathways are implicated in the secretion of polysialic acid Group 2 CPS-like polymers by pathogenic species (30). Alternatively, alginate, cellulose, and poly-*N*-acetyl-d-glucosamine (PNAG) polymers are produced via synthase-dependent schemes in which monosaccharide addition in the cytoplasm by an integral IM synthase results in export of the polymer by a similar amount from the cell surface; polymer transport through the periplasm is mediated by a protein scaffold containing tetratricopeptide repeats followed by translocation across the OM through an integral OM β-barrel porin structure (31). While similarities exist between Wzx/Wzy- and ABC transporter-dependent pathways (e.g., PCP and OPX proteins), no schematic crossover with synthase-dependent pathway proteins has been identified.

OPX-family proteins in Wzx/Wzy- and ABC transporter-dependent pathways are portrayed as forming a contiguous oligomeric channel for polymer secretion through the periplasm and across the OM (1). All OPX proteins share a conserved periplasmic N-terminal Poly_export domain (Pfam: PF02563), followed by at least one copy of a soluble ligand-binding β-grasp (SLBB) domain (Pfam: PF10531) (32, 33) that is predicted to interact with the sugar polymer in the periplasm (34). OPX protein domain architecture diverges at this point. In the prototypic E. coli Group 1 CPS OPX protein Wza (Wza*_Ec_*)—the only OPX protein with a solved 3D structure—after one Poly_export and two SLBB domains, the protein contains a C-terminal Wza_C domain (Pfam: PF18412), which forms a 35-residue OM-spanning amphipathic α-helix (35) (Fig. 1A). Elucidation of the Wza*_Ec_* structure (PDB: 2J58) was revolutionary, as it represented the first identification of an OM-spanning α-helix, with 8 copies of the Wza_C domain forming a pore-like structure through which it was proposed that secreted polysaccharides exit the cell (35, 36) (Fig. 1A). Conversely, OPX proteins from Group 2 CPS pathways usually contain a C-terminal Caps_synth_GfcC (Pfam: PF06251, formerly DUF1017) module (33), structurally similar to the stand-alone GfcC protein (PDB: 3P42) from Group 4 CPS pathways (32). GfcC contains two Wza*_Ec_*-like SLBB domains and a C-terminal amphipathic α-helix (spanning the final 21 residues of the protein, 40% shorter than that of Wza*_Ec_*) (37). This overall OPX architecture is typified by the E. coli Group 2 CPS-pathway KpsD (KpsD*_Ec_*) (32). Though it is uncertain if the KpsD*_Ec_* C terminus is able to span the OM, KpsD*_Ec_* epitopes have been detected at the cell surface using anti-KpsD*_Ec_* antibodies (38). Other OPX proteins have been shown to either (i) contain considerable yet uncharacterized protein sequences following their most C-terminal identified domain or (ii) be considerably shorter than either Wza*_Ec_* or KpsD*_Ec_*, with architecture beyond the Poly_export and SLBB domains largely absent (33). However, for OPX proteins lacking Wza_C domains, the manner by which the respective secreted polymers traverse the OM remains an open question.

Herein, we reveal the WzaX/S/B OPX proteins from the respective M. xanthus EPS/MASC/BPS pathways to contain N-terminal Poly_export-SLBB architecture but lack C-terminal Wza_C domains. Comparative genomics analyses reveal this architecture to be the most common among three OPX protein structural classes across all bacteria. However, a conserved β-barrel protein (MXAN_7418/3226/1916) is encoded beside *wzaX*/*wzaS*/*wzaB* in the respective M. xanthus EPS/MASC/BPS biosynthesis clusters. Fold-recognition and deep-learning analyses reveal these adjacently encoded proteins to be 18-stranded integral OM β-barrels with structural homology to the porin required for synthase pathway-dependent PNAG secretion across the OM. In turn, such β-barrel proteins are shown to be encoded near numerous genes representing all three OPX protein structural classes in diverse Gram-negative bacteria. The interior electrostatics of the M. xanthus β-barrels match known properties of their associated polymers, and deletion of the MXAN_7418 β-barrel is shown to compromise EPS secretion; this is consistent with the previously unknown reason for MXAN_3226-dependent MASC secretion (14). Taken together, these data support a novel and widespread secretion paradigm for Wzx/Wzy- and ABC transporter-dependent pathways in which those containing non-OM-spanning OPX components instead utilize β-barrel porins to mediate polymer translocation across the OM.

## RESULTS

### The M. xanthus OPX proteins WzaX, WzaS, and WzaB lack an OM-spanning α-helix domain.

Each of the WzaX/S/B OPX proteins is essential for the secretion of its respective EPS/MASC/BPS polymer in M. xanthus (3, 14). However, as WzaX/S/B have 44/50/46% (respectively) fewer residues than Wza*_Ec_*, we sought to better understand the structural implications of this difference. Fold-recognition analysis of WzaX/S/B via HHpred revealed 100/100/99.9% probability matches (respectively) to the N-terminal half of the high-resolution Wza*_Ec_* X-ray crystal structure (35), with Wza*_Ec_*-based WzaX/S/B tertiary structure modeling (via MODELLER) revealing the M. xanthus OPX proteins to be missing the 2nd SLBB domain and the OM-spanning α-helix of Wza*_Ec_* (Fig. 1B). The absence of such an OM-spanning domain is consistent with the lack of WzaX/S/B detection in proteomic analyses by the Søgaard-Andersen lab of OM vesicle (OMV) and biotinylated surface-protein samples (39), despite the constitutive expression of the *wzaX/S/B* genes throughout the M. xanthus life cycle (40, 41).

### OPX proteins constitute three distinct structural classes.

**FIG 2:**
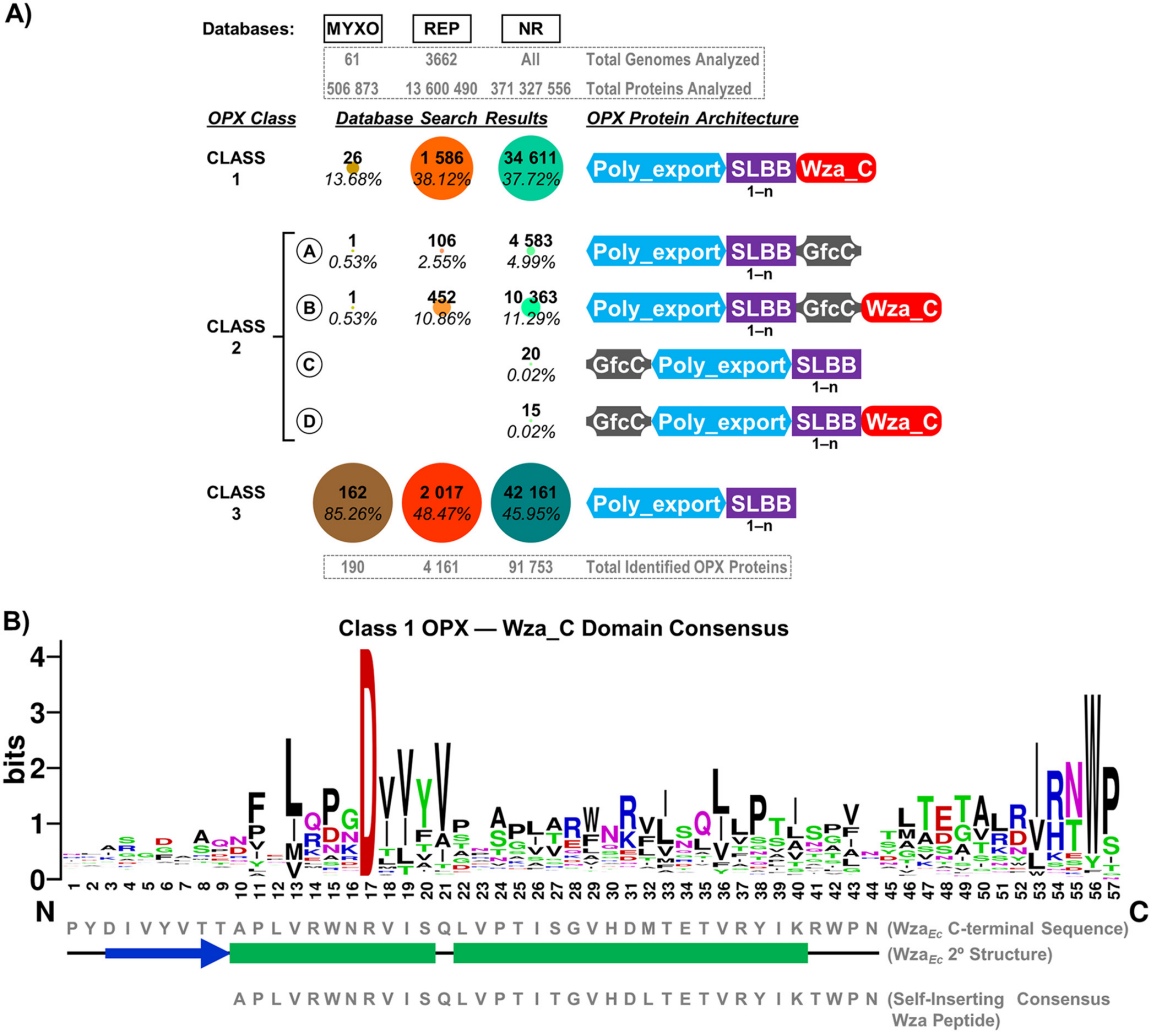
Structural diversity of OPX proteins. (A) Domain organization and abundance of bacterial OPX protein classes identified in the myxobacterial (MYXO: 61 genomes, 506,873 proteins analyzed), representative (REP: 3,662 genomes, 13,600,490 proteins analyzed), and NCBI nonredundant (NR: 371,327,556 proteins analyzed) databases. The Poly_export (PF02563), SLBB (PF10531), Wza_C (PF18412), and Caps_synth_GfcC (PF06251) Pfam domains were used to scan all three databases, followed by fold-recognition analysis using HHpred against the 3D X-ray crystal structures of Wza*_Ec_* (PDB: 2J58) and GfcC (PDB: 3P42). The number of repeated domains is indicated under each domain depiction. The number of OPX hits (bold) for a specific class and the proportion of these hits relative to all others in the particular database (italics) are indicated. (B) Sequence logo of the consensus amino acids constituting the OM-spanning α-helix based on a multiple-sequence alignment of 1,586 class 1 OPX proteins. The region of sequence alignment with Wza*_Ec_* is indicated and depicted with the associated secondary structure from the Wza*_Ec_* X-ray crystal structure (PDB: 2J58) (35). Also depicted is the region of sequence alignment with a previously published optimized Wza_C synthetic peptide (based on 94 close Wza*_Ec_*-related homologues) capable of spontaneously inserting into lipid bilayers and self-assembling into stable α-barrel pores (64). The positions of observed α-helices (boxes) and β-strands (arrows) are indicated.

To determine if the absence of the Wza_C domain was an aberration confined to the subset of OPX proteins from M. xanthus under study, we first performed a massive comparative genomics analysis using profile-based homology searches via hmmscan across three different data sets—(i) 61 myxobacterial genomes (MYXO) (see Table S1 in the supplemental material), (ii) 3,662 complete representative genomes (REP) (Table S2), and (iii) the nonredundant (NR) NCBI database (371,327,556 proteins at 100% identity as of 10 June 2021) (Table S3)—to identify encoded OPX proteins. All three databases were scanned against PF02563 (Poly_export), PF10531 (SLBB), PF18412 (Wza_C), and PF06251 (Caps_synth_GfcC; referred to here as GfcC) as our query domains. These profile-based analyses identified diverse putative OPX homologues that could be divided into three distinct classes according to their domain architecture (Fig. 2A). The first set of OPX proteins was found to contain Poly_export–SLBB_(1–14)_ architecture (i.e., 1 to 14 occurrences of SLBB domains) ending with a C-terminal OM-spanning Wza_C domain, similar to Wza*_Ec_*, and was assigned the designation “Class 1” (Fig. 2A). The second set of OPX proteins was found to possess Poly_export-SLBB_(1–6)_-GfcC architecture similar to KpsD*_Ec_*, ending with or without a C-terminal OM-spanning Wza_C domain, and was assigned the designation “Class 2.” Finally, the third set, containing the most OPX proteins, was found to contain only Poly_export–SLBB_(1–7)_ architecture lacking either a Wza_C or GfcC domain; these hits were designated “Class 3”; however, many of these initial hits were found to contain additional amino acids that may have remained uncharacterized following sequence-based domain detection. To probe these partially characterized hits in more detail, all identified OPX proteins were analyzed further via fold recognition using HHpred to identify matches with more remote sequence homology but conserved structural properties. These analyses resulted in the reclassification of several original Class 3 hits to either Class 1 or Class 2.

Incidentally for Class 1 OPX proteins, while the secondary structure was conserved, considerable sequence variation was detected within certain regions of the putative OM-spanning Wza_C domains, with this domain extending up to 48 residues in length (compared to 35 residues in Wza*_Ec_*) (Fig. 2B). As per the MYXO/REP/NR databases, 13.7/38.1/37.7% of OPX proteins possess Class 1 Wza*_Ec_*-like organization with a putative OM-spanning C-terminal α-helix (Fig. 2A; Tables S1, S2, and S3). Class 1 OPX proteins (median length, 378 amino acids [aa]) were largely (1,233/1,586; ~78%) predicted to be lipoproteins with Sec/SPII signal sequences. Based on species-level PSORTdb classification (42), the REP database contains 698 Gram-positive and 1,381 Gram-negative organisms. Our analysis revealed that class 1 OPX proteins are encoded by many Gram-negative bacteria (639/1,381 genomes; ~46%), whereas these proteins were completely absent in Gram-positive species (Table S2B).

Our MYXO/REP/NR database comparative genomic analysis revealed that Class 2 OPX proteins could be further divided into four subclasses. Proteins belonging to Class 2A contain Poly_export-SLBB_(1–_*_n_*_)_-GfcC architecture, whereas those assigned to Class 2B possess Poly_export-SLBB_(1–_*_n_*_)_-GfcC–Wza_C architecture ending with an OM-spanning α-helical domain. Classes 2C and 2D are variations of Classes 2A and 2B (respectively), where a GfcC domain precedes the Poly_export domain; however, only 20 Class 2C and 15 Class 2D proteins were identified across the entire NR database.

Class 2A OPX proteins (median length, 605 aa) constitute 0.5/2.6/5.0% of all OPX proteins identified in the MYXO/REP/NR databases; they are encoded in only ~5% (66/1,381 genomes) of Gram-negative bacteria and are completely absent among Gram-positive species (Table S2B). Similarly, Class 2B OPX proteins (median length, 824 aa) represent 0.5/10.9/11.3% of all identified MYXO/REP/NR-database OPX proteins (Fig. 2A; Tables S1, S2, and S3), with most (375/452, ~83%) found to possess standard Sec/SPI secretory signal peptides. Class 2B OPX proteins have representation in only ~18% (243/1,381 genomes) of Gram-negative organisms and are absent in Gram-positive bacteria (Table S2B).

**FIG 3:**
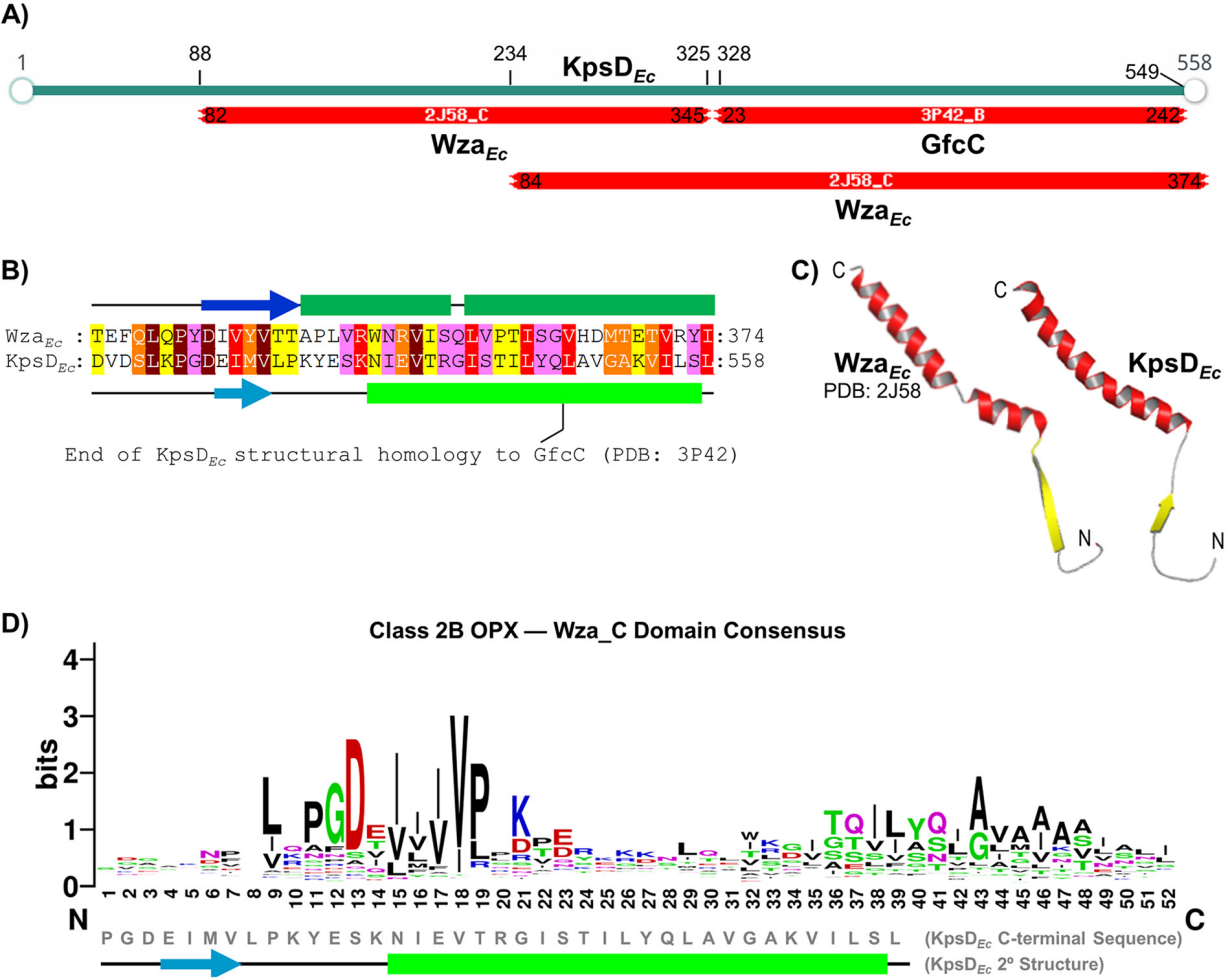
Structural homology between Wza*_Ec_* and KpsD*_Ec_*. (A) Fold-recognition analysis of KpsD*_Ec_* (via HHpred) revealing C-terminal structural homology to GfcC (PDB: 3P42) as well as Wza*_Ec_* (PDB: 2J58). (B) Profile-based alignment of Wza*_Ec_* and KpsD*_Ec_* C-terminal sequences from panel A. Wza*_Ec_* α-helix (dark green cylinders) and β-strand (dark blue arrows) structures are depicted as per the 2J58 PDB entry. KpsD*_Ec_*-predicted α-helix (light green cylinders) and β-strand (light blue arrows) secondary structures are indicated as per PSIPRED analysis. Aligned residues are colored according to JalView conservation score (out of 10). Maroon, 10; red, 9; orange, 8; yellow, 7; pink, 6. Scores of 5 or less have been omitted to improve clarity of the figure. The end of KpsD*_Ec_* structural homology with the stand-alone GfcC protein is indicated as per a previous report (32). (C) Tertiary structure model of the KpsD*_Ec_* C terminus based on structural alignment with Wza*_Ec_* as indicated in panel B. N and C termini of the displayed peptide are indicated. (D) Sequence logo of the consensus amino acids constituting the putative OM-spanning α-helix based on a multiple-sequence alignment of 452 class 2B OPX proteins. The region of sequence alignment with KpsD*_Ec_* is indicated, along with the predicted KpsD*_Ec_* secondary structure. The position of predicted α-helices (boxes) and β-strands (arrows) is indicated.

Class 2B architecture is typified by KpsD*_Ec_*. Consistent with a previous report (32), fold-recognition analysis of KpsD*_Ec_* revealed that most of its N terminus is structurally homologous to Wza*_Ec_*, while the bulk of its C terminus is a structural match to the standalone GfcC protein (Fig. 3A). However, the extreme C terminus of KpsD*_Ec_*—i.e., the portion of KpsD*_Ec_* surpassing the end of structural homology with the GfcC D4 α-helix—was found to have considerable structural homology with the most C-terminal region of Wza*_Ec_*, including a 25-residue tract with α-helical propensity matched to the OM-spanning α-helical tract of Wza*_Ec_* (Fig. 3B and C). A similarly extended C-terminal α-helix was found throughout the Class 2B OPX hits identified here, with considerable variation in certain regions of its sequence, and extending to 38 residues (compared to 25 residues in KpsD*_Ec_*) (Fig. 3D). This observation supports the notion that a part of KpsD*_Ec_* (and, by extension, Class 2B OPX proteins) may indeed be able to span the OM and access the cell surface.

Finally, Class 3 OPX proteins (median length, 256 aa) with only Poly_export-SLBB_(1–_*_n_*_)_ architecture represent a majority (~85/49/46%) of OPX proteins identified across the MYXO/REP/NR databases (Fig. 2A; Tables S1, S2, and S3). Almost 50% are predicted lipoproteins (Sec/SPII signal sequences), while ~30% are secreted proteins (Sec/SPI signal sequences). Within the MXYO data set, Class 3 OPX proteins were encoded in all 61 myxobacterial organisms, without any exception, in the range of 1 to 4 proteins (Table S1). Expectedly, our analysis detected Class 3 OPX proteins in Gram-negative bacteria (600/1,382 genomes, ~43%), but also, intriguingly, in various Gram-positive organisms (52/699 genomes, ~7%) (Table S2B). Of note, the proportions of each class of OPX protein detected in the REP database were highly reflective of those found in the NR database (Fig. 2A), reinforcing the utility and applicability of the REP database.

### Molecular phylogenetics suggests the coevolution of three OPX protein classes.

**FIG 4:**
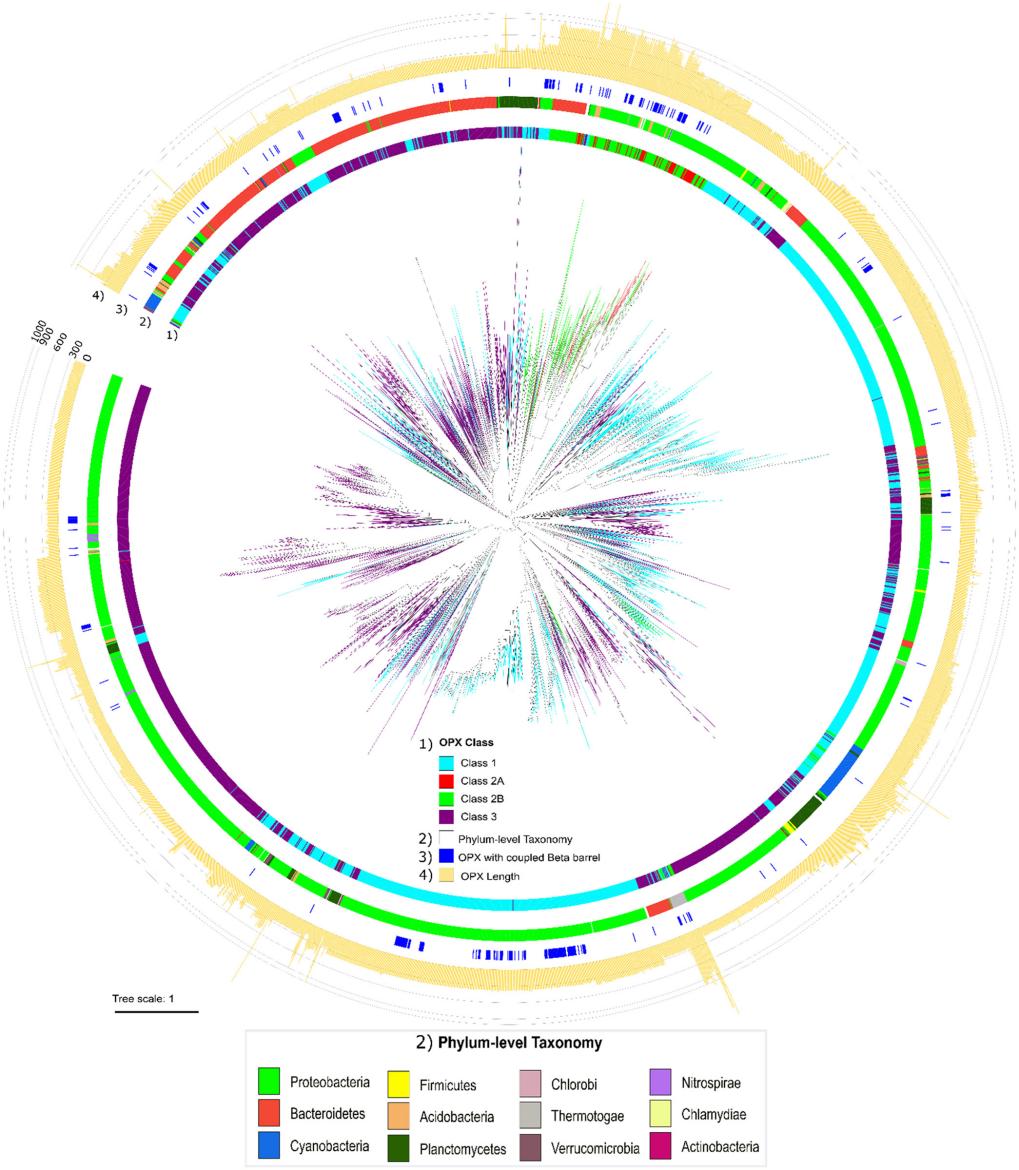
Phylogenetic tree of OPX proteins. Sequence alignment of all Poly_export domains as identified in 4,161 OPX proteins in the REP data set was used to generate a maximum likelihood phylogenetic tree. The tree also represents OPX protein classification (innermost tree branches and ring 1), their respective taxonomy at the phylum level (ring 2), the presence of nearby β-barrels (ring 3), and the length of each OPX protein (ring 4) accordingly (from inside to outside) for effective visualization.

Given that all OPX proteins among the three classes have a conserved Poly_export domain, this can be utilized as a phylogenetic marker to study relationships between the various hits. Therefore, based on the hmmscan results, we extracted the location of the Poly_export domain from REP data set hits, aligned those sequences using MUSCLE, and generated a maximum likelihood phylogeny (Fig. 4). This phylogenetic tree revealed that Class 1 and Class 3 OPX proteins are widely interspersed with each other in all taxonomic clades, suggesting that proteins from these two classes may have coevolved by losing/gaining the Wza_C segment in closely related organisms. However, Class 2A and Class 2B OPX homologues share nearby sister clades in the phylogeny and are distant from Class 1 and Class 3 OPX homologues. Based on closer distance and representative architecture (Poly_export-SLBB_(1–6)_-GfcC), this phylogenetic tree denotes that Class 2 OPX proteins are distinct from Class 1 and Class 3 and that both subclasses (Class 2A and Class 2B) might have coevolved by losing/gaining their respective Wza_C segments while maintaining their overall architecture.

### OPX protein lengths and classes in Gram-negative bacteria are not linked to periplasm thickness.

**FIG 5:**
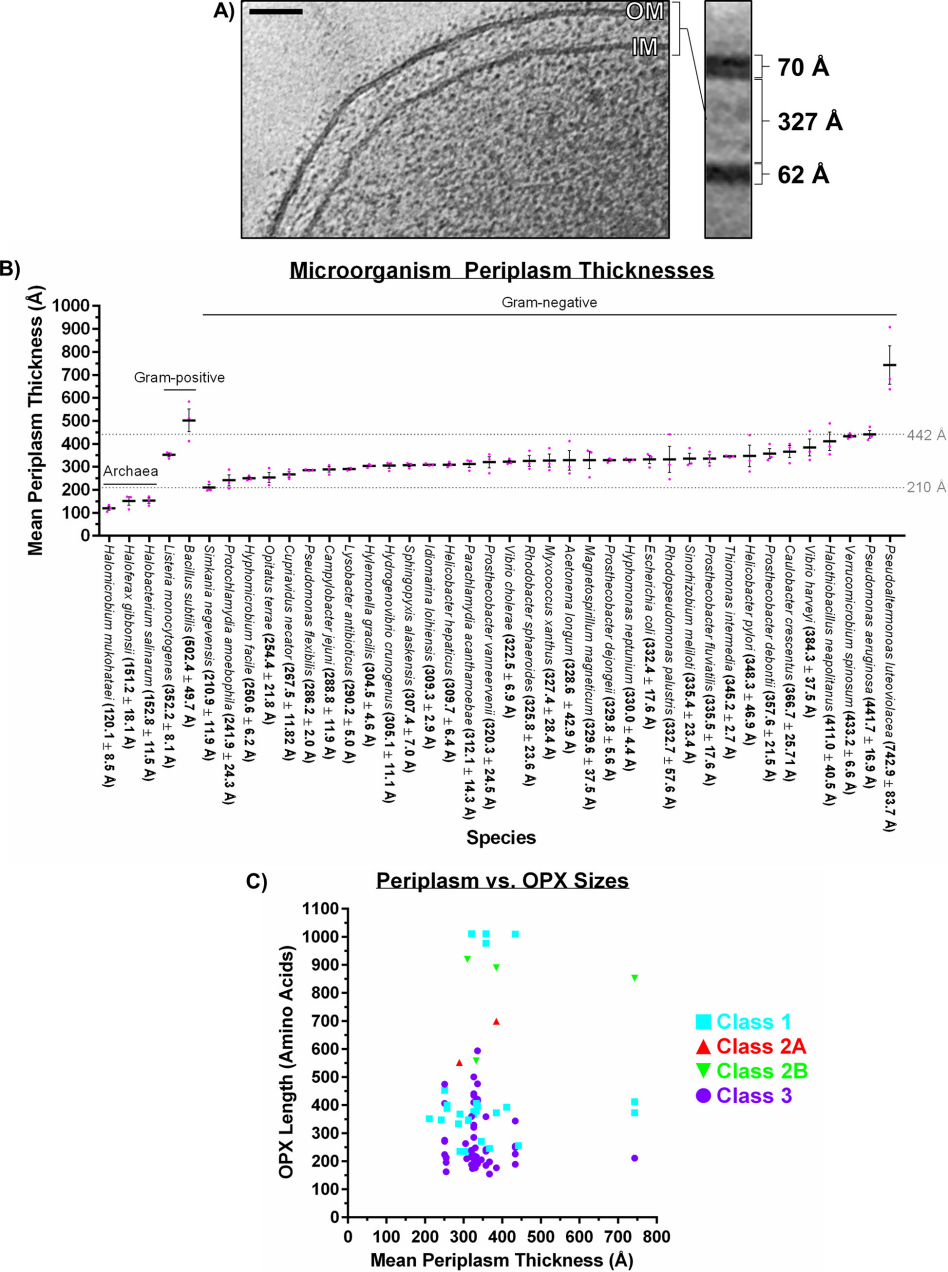
Cell envelope ultrastructure in Gram-negative bacteria. (A) Cryo-electron tomography slice of an M. xanthus cell showing the IM, OM, and intervening periplasmic space and their respective measured thicknesses. (Adapted from reference 46 with permission of the publisher.) (B) Comparison of means (black bar) for periplasmic distances in 40 microbial species (± standard error of the mean [SEM]). Individual replicate measurements are indicated (magenta dots). Mean values (±SEM) are also listed after each organism name. IM–peptidoglycan and IM–S-layer thickness in several Gram-positive bacteria and *Archaea* (respectively) were provided for reference. Data from organisms with increasing mean periplasmic thickness values are depicted from left to right, grouped according to Gram-negative, Gram-positive, or *Archaea* organism designation. Tomograms were downloaded from the Caltech Electron Tomography Database (https://etdb.caltech.edu/) (47). (C) Scatterplot of mean periplasm thickness values plotted against the length of each OPX protein from the same organism. Data points are colored according to the class of OPX protein assigned here. No correlation was detected between periplasmic thickness and (i) overall OPX protein length (Pearson coefficient, 0.1450; Spearman coefficient, 0.05884; calculated over 92 data pairs), (ii) Class 1 OPX hits (Pearson coefficient, 0.07250; Spearman coefficient, 0.2318; calculated over 33 data pairs), or (iii) Class 3 OPX hits (Pearson coefficient, −0.1127; Spearman coefficient, −0.1242; calculated over 53 data pairs).

In E. coli, the integral OM Class 1 OPX protein Wza*_Ec_* is proposed to form a complex with the integral IM Wzc*_Ec_* PCP protein that creates a contiguous periplasm-spanning channel for polymer export (43) (Fig. 1A). However, the reported periplasmic distance to be covered in such a scenario differs depending on the methods used to obtain ultrastructural measurements of the bacterial cell envelope. This is exemplified by different studies of E. coli in which cryo-electron microscopy was used to obtain projection images with which to obtain a measurement of periplasmic distance; one investigation used cells that were cryo-sectioned (a process known to introduce sample distortions due to cutting) into ~50 nm-thick slices (44), while the other used intact cells (>500 nm thick) without sectioning (in which the projection of large, curved membrane may introduce complications/smearing) (45). Given these potential pitfalls, we elected to harness the power of cryo-electron tomography, a technique that images intact cells without the need for sectioning (thus negating any cutting-induced sample distortions) and which allows for analysis of computationally reconstructed thin sections through the cell (thus preventing the problem of projecting the entirety of a thick cell on one image for analysis) (46). To therefore gain an understanding of the relationship between the subcellular architecture of M. xanthus and the role of the WzaX/S/B Class 3 OPX proteins, we compared the sizes of various cellular compartments and structures from previous cryo-electron tomography thin sections (representing a thickness of ~10 nm) of the M. xanthus envelope (46). This revealed the M. xanthus OM to have an average thickness of 69.8 ± 1.8 Å, compared to the average thickness of 62 ± 1.6 Å for its IM, with a mean intermembrane periplasm thickness of 327 ± 28.4 Å (Fig. 5A).

Given the enrichment of Class 1 OPX proteins in certain bacterial genera and different median sizes for each OPX protein class (Table S4), we next examined whether OPX protein size was associated with periplasm thickness in a given bacterium. We first measured the distance between the IM and OM at lateral positions in cryo-electron tomograms of cells (downloaded from the Caltech Electron Tomography Database [https://etdb.caltech.edu/]) (47) from an additional 34 species of Gram-negative bacteria, revealing a range of periplasm thicknesses of between 210 and 442 Å for all but one strain (Fig. 5B). Importantly, (i) our measurement of 332.4 ± 17.6 Å for the size of the E. coli periplasm (from ~10-nm-thick reconstructed sections) is in line with that previously described from ~50 nm-thick cut sections (44), and (ii) our data are consistent with this previous report showing the periplasm of Pseudomonas aeruginosa to be larger than that of E. coli (44) (Fig. 5B). For any of the species from the Caltech Electron Tomography Database with herein-identified OPX proteins (Tables S1, S2, and S3), we next compared the mean thickness of the periplasm with the length of the OPX protein(s) in each system. However, no correlation between these variables was detected across all OPX proteins in this analysis, nor specifically, within Class 1 or Class 3 OPX hits (Fig. 5C).

### WzaX/S/B are genomically paired with 18-stranded β-barrel proteins.

Given the lack of WzaX/S/B OM-spanning domains (Fig. 1B), we sought to identify candidate proteins that could permit export of synthesized EPS/MASC/BPS polymers across the M. xanthus OM. Through our previous analyses of the EPS/MASC/BPS biosynthesis clusters, WzaX (MXAN_7417)/WzaS (MXAN_3225)/WzaB (MXAN_1915) were found to be encoded next to (i.e., 3 bp upstream/65 bp upstream/23 bp downstream) MXAN_7418/3226/1916 (respectively), with this synteny conserved for the majority (115/162, ~71%) of myxobacterial Class 3 OPX proteins (3), supporting the notion that the latter three proteins are important for each respective pathway.

Previous bioinformatic analysis had already predicted MXAN_1916 to be an integral OM β-barrel protein (48). Evolutionary-coupling analyses predicted the presence of 18 principal β-strands for MXAN_7418/3226/1916 (Fig. S1A, S2A, and S3A), with fold recognition revealing structural homology of the C-terminal 76 to 86% of MXAN_7418/3226/1916 to the integral OM β-barrel module of PgaA (PgaA_βb_, PDB: 4Y25) (49), at 98.9/99.4/99.2% probability. PgaA is the OM porin responsible for secretion of synthase-dependent PNAG; it contains multiple periplasmic tetratricopeptide repeats at its N terminus, followed by a 16-stranded integral OM β-barrel domain (31, 49). In MXAN_7418/3226/1916, the PgaA_βb_-like module is extended by two integral OM β-strands at the N terminus, suggesting that these proteins do indeed have the propensity to form 18-stranded β-barrels (Fig. S1B, S2B, and S3B).

For the 162 Class 3 OPX proteins identified across 61 myxobacterial genomes (with Poly_export-SLBB_1–2_ architecture), most (115/162, ~71%) were found to be encoded near an extended PgaA_βb_-like protein, whereas the 26 Class 1 (with Poly_export-SLBB_1–2_-Wza_C organization) and 2 Class 2 OPX proteins were not encoded near any such β-barrel protein (Table S1). We again expanded our analysis beyond M. xanthus to determine whether the presence of β-barrel porins was a common occurrence in pathways containing OPX proteins. Intriguingly, in E. coli, the *gfcABCDE-etp-etk* Group 4 CPS cluster encodes the Class-1 OPX protein GfcE (formerly YccZ/Wza_22min_) and the GfcD (formerly YmcA) protein (50). The separate *yjbEFGH* (paralogous to *gfcABCD*) operon implicated in polysaccharide secretion encodes the GfcD-like protein YjbH (51). Both GfcD and YjbH were recently identified to be part of a novel class of OM proteins predicted to contain two β-barrels formed by the same polypeptide (52). Herein, fold-recognition analysis revealed the N-terminal halves to be matches to the β-barrel amyloid transporter FapF from Pseudomonas, whereas the C-terminal halves (GfcD_Cterβb_ and YjbH_Cterβb_) possessed primary and tertiary structural homology to the PNAG porin module described above (Fig. S4A and S4B). This double-barrel arrangement was supported by AlphaFold-generated deep-learning structure models for both full-length GfcD and YjbH (Fig. S4C).

To probe for the presence of similar β-barrels encoded near other OPX proteins, we used sequence homology searches (BLAST and HMMER) to examine the genomic context (± 10 genes) of the various OPX proteins identified in the REP and MYXO data sets, beginning with the MXAN_7418/3226/1916 sequences. Given the homology of the above-described proteins to PgaA, we added the PgaA_βb_, GfcD_Cterβb_, and YjbH_Cterβb_ sequences as well. In addition, the β-barrel sequences of BcsC (BcsC_βb_, PDB: 6TZK) (53) and AlgE (PDB: 4AFK) (54) were also included, given their porin functions in synthase-dependent cellulose and alginate production, respectively (31). We also included the sequence of Wzi (PDB: 2YNK) lacking the α-helical-plug domain (Wzi_βb_), as this is an 18-stranded β-barrel linked with polysaccharide biosynthesis clusters (29) (Fig. S5).

Altogether, this analysis detected 365 β-barrel query homologues encoded near 344 OPX proteins of all three classes (Table 1). For β-barrel query sequences from proteins with additional native β-barrel and/or α-helical domains (Fig. S5), certain homologues were identified that correspond to the various full-length proteins, while others exclusively matched the polysaccharide porin modules (Table 1). These latter data are particularly significant for Wzi, as 28 homologues were a match to only Wzi_βb_ (i.e., no plug domain) (Fig. S5, Table 1), reinforcing their candidacies as trans-OM export β-barrels; intriguingly, HHpred analysis of these 28 hits also revealed many with strong similarity to DUF6029, ascribing a potential polysaccharide secretion role to this heretofore uncharacterized protein domain. MXAN_7418/3226/1916 homologues were only found to be encoded in myxobacterial genomes, with the presence of these β-barrels linked exclusively to nearby myxobacterial Class-3 OPX proteins.

**TABLE 1:** β-barrels identified to be syntenic with OPX genes in the MYXO and REP data sets

β-Barrel query template^*a*^	Total β-barrel homologues detected near OPX genes	β-Barrel homologues detected near Class 1 OPX genes	β-Barrel homologues detected near Class 2A OPX genes	β-Barrel homologues detected near Class 2B OPX genes	β-Barrel homologues detected near Class 3 OPX genes
MXAN_7418	MXAN_7418: 6	−^*b*^	−	−	6
MXAN_3226	MXAN_3226: 9	−	−	−	9
MXAN_1916	MXAN_1916: 12	−	−	−	12
PgaA_βb_	PgaA: 8	3	−	1	4
	PgaA_βb_: 2	2	−	−	−
GfcD_Cterβb_	GfcD: 66	44	4	18	−
	GfcD_Cterβb_: 2	−	1	1	−
YjbH_Cterβb_	YjbH: 79	55	5	19	−
	YjbH_Cterβb_: 3	1	1	1	−
BcsC_βb_	BcsC: 5	1	−	−	4
	BcsC_βb_: −	−	−	−	−
AlgE	AlgE: 14	6	−	2	6
Wzi_βb_	Wzi: 133	44	9	48	32
	Wzi_βb_: 28	5	1	14	8

a See Fig. S5 for query template structures.

b −, No detected occurrence.

Together, these data reveal intriguing architectural similarities between β-barrel porin modules from synthase-dependent polymer export pathways and those implicated in myxobacterial Wzx/Wzy-dependent secretion, as well as analogous or ABC transporter-dependent pathways in diverse bacteria, all previously unreported associations.

### WzpX/S/B are respective integral-OM β-barrel EPS/MASC/BPS-pathway porins.

**FIG 6:**
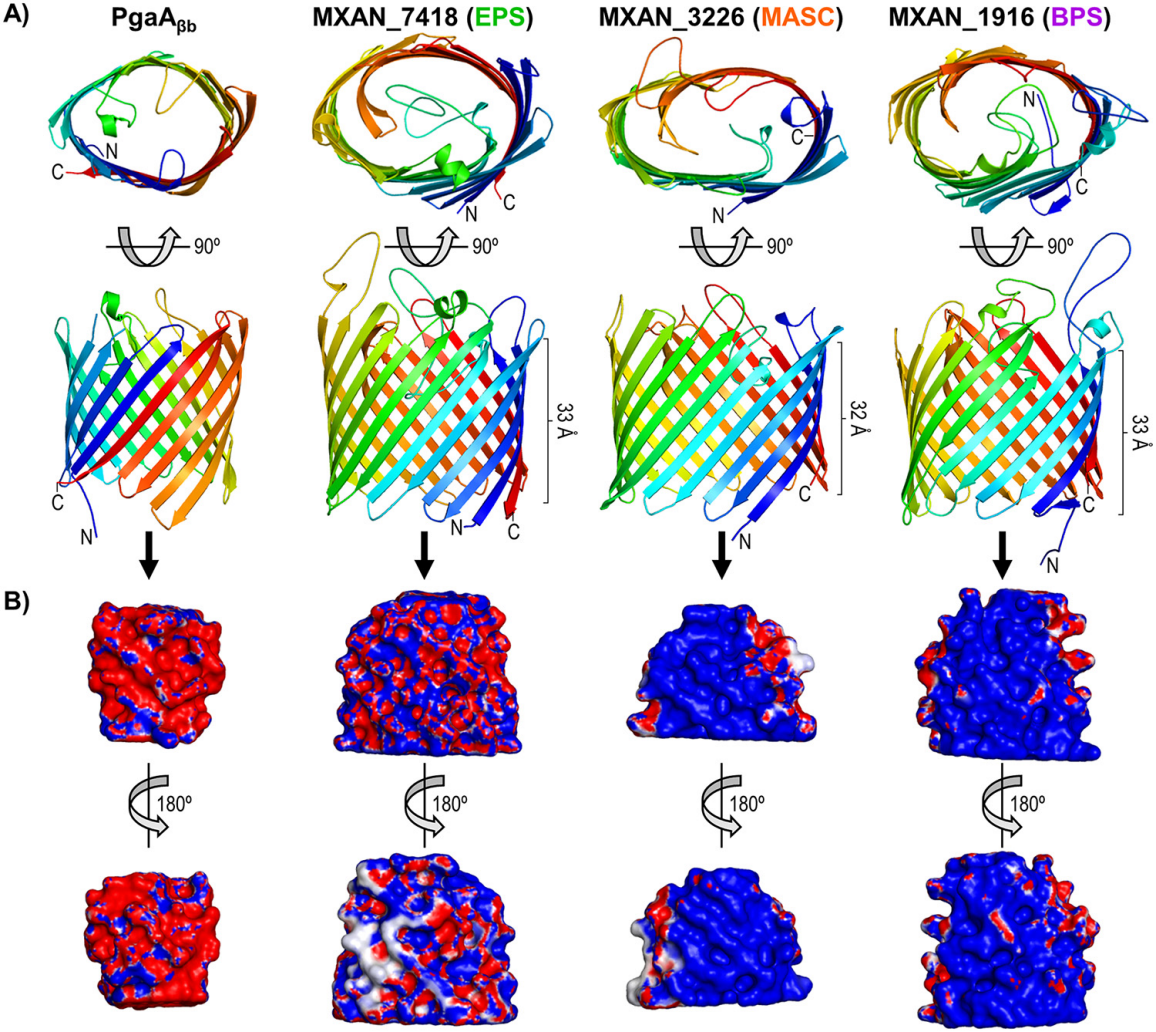
OPX-companion β-barrel model structures. (A) Tertiary structure models (top and front views) for MXAN_7418, MXAN_3226, and MXAN_1916, generated using deep learning via AlphaFold as well as the PgaA C-terminal domain (aa 513 to 807) X-ray crystal structure (PDB: 4Y25) (49). Structures are colored with a spectrum, from the N terminus (blue) to the C terminus (red), and depicted with smooth loops. (B) Front and back views of the interior spaces of the β-barrels depicted in panel A overlaid with the electrostatic character of the residues contacting the lumenal volume, generated via HOLLOW (77). Surfaces are colored according to charge, from blue (positive, +5 kT/e) to white (uncharged/hydrophobic), to red (negative, −5 kT/e) (where “k” is Boltzmann’s constant, “T” is the temperature (in Kelvin), and “e” is the electron charge).

To examine the structural suitability of the WzaX/S/B β-barrels for EPS/MASC/BPS translocation in the absence of a full template structure, we employed the AlphaFold deep-learning approach to generate a tertiary structure model for each protein. AlphaFold employs evolutionarily coupled amino acid data and templates with structural homology to fold a polypeptide using an iterative process (55). Consistent with the above-described data (Fig. S1, S2, and S3), MXAN_7418/3226/1916 were all predicted to form 18-stranded β-barrels with sizeable central cavities, with respective barrel heights of 33, 32, and 33 Å (Fig. 6A). As molecular dynamics simulations calculate the hydrophobic thickness of asymmetric OM bilayers to be ~40% of their total solvated thickness (56), based on our measured M. xanthus OM thickness of 69.8 ± 1.8 Å (Fig. 5A), an approximated hydrophobic thickness of ~28 Å would indeed be traversable by the MXAN_7418/3226/1916 structures.

We subsequently used HOLLOW to probe the lumenal volume of the EPS/MASC/BPS-cluster β-barrels via filling of the internal space with dummy atoms to generate a cast of the void space, after which the electrostatic potential of the contacting β-barrel surface was overlaid. To validate this approach, we first probed the PgaA_βb_ template interior, revealing a highly anionic interior (Fig. 6B), consistent with passage of the cationic PNAG polymer through the PgaA_βb_ lumen. BPS was previously discovered to be a randomly acetylated anionic repeating tetrasaccharide, with the distal three sugars of each repeat constituted by mannosaminuronic acid (ManNAcA) units (3). Therefore, the cationic charge character of the MXAN_1916 lumen (Fig. 6B) is indeed suitable for passage of anionic BPS. While the chemical structures or exact compositions of MASC or EPS are not known, spore coat material was found to contain GalNAc chains with potential Glc and glycine decorations (14). As the interior of the MXAN_3226 β-barrel is cationic (Fig. 6B), this suggests that MASC may have a net-anionic charge, as contributed via as yet unidentified components. EPS composition has been probed across four investigations (3, 57–59), with Ara, Gal, GalNAc, Glc, GlcN, GlcNAc, Man, ManNAc, Rha, and Xyl identified (depending on the publication); however, none of these sugars are highly charged, which is consistent with the more neutral character of the MXAN_7418 interior (compared to that of MXAN_1916 or MXAN_3226) (Fig. 6B).

**FIG 7:**
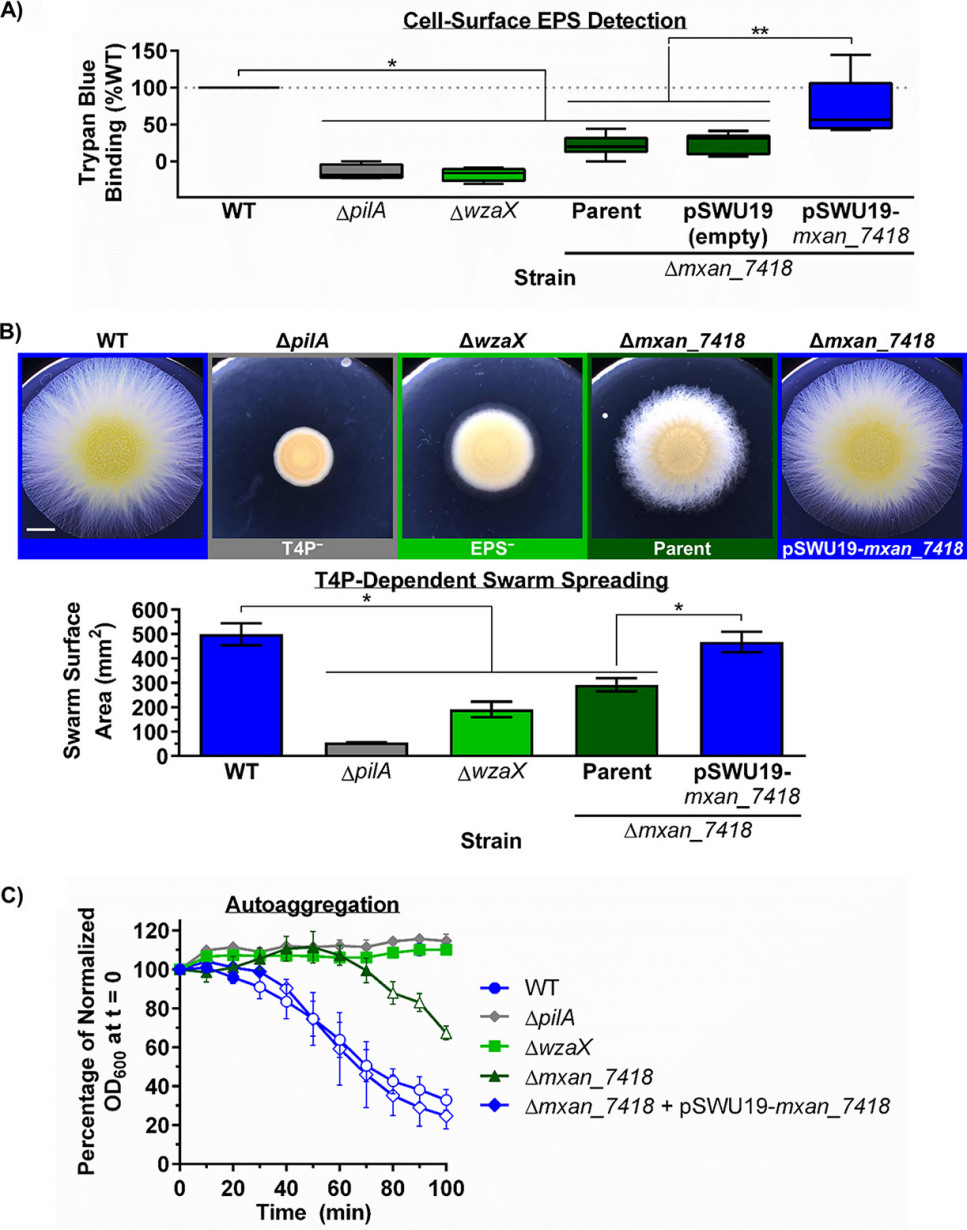
MXAN_7418 mediates EPS secretion in M. xanthus. (A) Boxplots of trypan blue dye retention for M. xanthus DZ2 WT (*n* = 14), Δ*pilA* (*n* = 4), Δ*wzaX* (*n* = 6), Δ*mxan_7418* (parent strain, *n* = 13), Δ*mxan_7418*+pSWU19 (empty, *n* = 6), and Δ*mxan_7418*+pSWU19-*mxan_7418* (*n* = 5) to probe cell-surface EPS levels. The lower and upper boundaries of the boxes correspond to the 25th and 75th percentiles, respectively. The median (line through center of boxplot) is indicated for each data set. Lower and upper whiskers represent the 5th and 95th percentiles, respectively. One asterisk (*) denotes data sets displaying statistically significant differences in means (*P* ≤ 0.0002) relative to a value of 100 (i.e., WT) as determined via two-tailed one-sample *t* test. The double asterisk (**) denotes statistically significant differences in distributions (*P* ≤ 0.0043) between the complemented strain Δ*mxan_7418*+pSWU19-*mxan_7418* and either Δ*mxan_7418* or Δ*mxan_7418*+pSWU19, as determined via two-tailed Mann-Whitney test. (B) T4P-dependent swarm expansion (CYE 0.5% agar, 72 h, 32°C) of EPS pathway β-barrel mutant and complemented M. xanthus strains. (Top) Stereoscope images of swarm expansion. Scale bar = 4 mm. (Bottom) Bar graph displaying the average (*n* = 4) swarm surface area ± SEM (mm^2^). The asterisk denotes statistically significant differences in mean values, as determined via unpaired two-tailed Student’s *t* test (*P* ≤ 0.0126). (C) Auto-aggregation profiles of M. xanthus DZ2 WT (*n* = 5), Δ*pilA* (*n* = 3), Δ*wzaX* (*n* = 6), Δ*mxan_7418* (*n* = 3), and Δ*mxan_7418*+pSWU19-*mxan_7418* (*n* = 4) cells resuspended in CYE rich medium at an initial OD_600_ of 1.0. Mean values are indicated at each time point ± SEM. Open plot points indicate statistically significant difference in the mean relative to Δ*wzaX* at a given time point. Closed plot points indicate no statistically significant difference in the mean relative to Δ*wzaX* at a given time point. Significance was evaluated via two-way analysis of variance (ANOVA) and Dunnett’s multiple-comparison test with a single pooled variance.

RNA sequencing (RNA-seq) analysis previously detected transcripts encoding MXAN_7418/1916 in vegetative cells, and MXAN_3226 in developmental cells, indicating that all three β-barrels are expressed over the course of the M. xanthus life cycle (40, 41). Furthermore, MXAN_1916 was detected in proteomic screens of biotinylated surface-exposed proteins, and MXAN_1916/3226 were both detected in OMV samples from vegetative cells (39). Importantly, MXAN_3226 was already shown by the Higgs lab to be essential for MASC secretion and myxospore development, but the reason was unknown at the time (14). To examine effects of β-barrel deletion on vegetative cells, we therefore generated a Δ*mxan_7418* deletion mutant strain and probed cell-surface EPS levels via trypan blue dye retention. We compared the dye-binding capacity of Δ*mxan_7418* cells versus that of EPS-pathway OPX^−^ (Δ*wzaX*) and T4P^−^ (Δ*pilA*) cells, both defective in EPS production. Relative to wild-type (WT) cells, the absence of the EPS pathway β-barrel resulted in an 80% median loss of trypan blue retention by Δ*mxan_7418* cells (Fig. 7A), indicating severely reduced cell surface EPS levels consistent with deficiencies in other EPS pathway mutant strains (3).

Compared to the baseline readings in the EPS pathway OPX mutant (Δ*wzaX*) strain, Δ*mxan_7418* cells bound marginally higher levels of trypan blue (Fig. 7A), consistent with EPS pathway β-barrel deficiency impacting polymer export to the cell surface, as opposed to polymer assembly and export being compromised in the EPS pathway OPX mutant. To test for residual quantities of cell surface EPS in Δ*mxan_7418*, we compared swarm expansion on solid medium and auto-aggregation in liquid medium; while multifactorial, both require T4P engagement with cell surface EPS. Relative to the WT, Δ*mxan_7418* swarm expansion was reduced, but not to the extent detected in Δ*wzaX* swarms (Fig. 7B). Consistent with previous findings, WT cells also steadily auto-aggregated and sedimented, whereas both Δ*pilA* and Δ*wzaX* cells did not (Fig. 7C); Δ*mxan_7418* cells remained in suspension analogous to Δ*wzaX* cells for ~75% of the assay, after which they began to slowly sediment (Fig. 7C), suggesting that cell surface EPS had eventually accumulated to a sufficient threshold to support T4P-mediated clumping in liquid. Together, these data suggest that while minimal amounts of EPS can reach the cell surface through as yet undetermined means (see Discussion), MXAN_7418 serves as the principal M. xanthus trans-OM EPS export conduit.

To probe for possible polar effects resulting from the Δ*mxan_7418* deletion, we complemented the EPS-pathway β-barrel deficiency via ectopic expression of MXAN_7418 (from the single-copy pSWU19 plasmid, driven by the *pilA* promoter) at the Mx8 *attB* phage-attachment site in the M. xanthus genome. This complementation resulted in a restoration of WT-like trypan blue dye binding indicative of the restored presence of cell surface EPS (Fig. 7A). In turn, the EPS-dependent phenotypes of swarm expansion and auto-aggregation were also restored to WT-like levels (Fig. 7B and C). These findings reinforce the notion that the cell surface EPS deficiency in the Δ*mxan_7418* mutant is precisely due to a lack of EPS secretion through a porin-like EPS pathway β-barrel (as opposed to downstream effects on precursor synthesis and/or polymer assembly).

**FIG 8:**
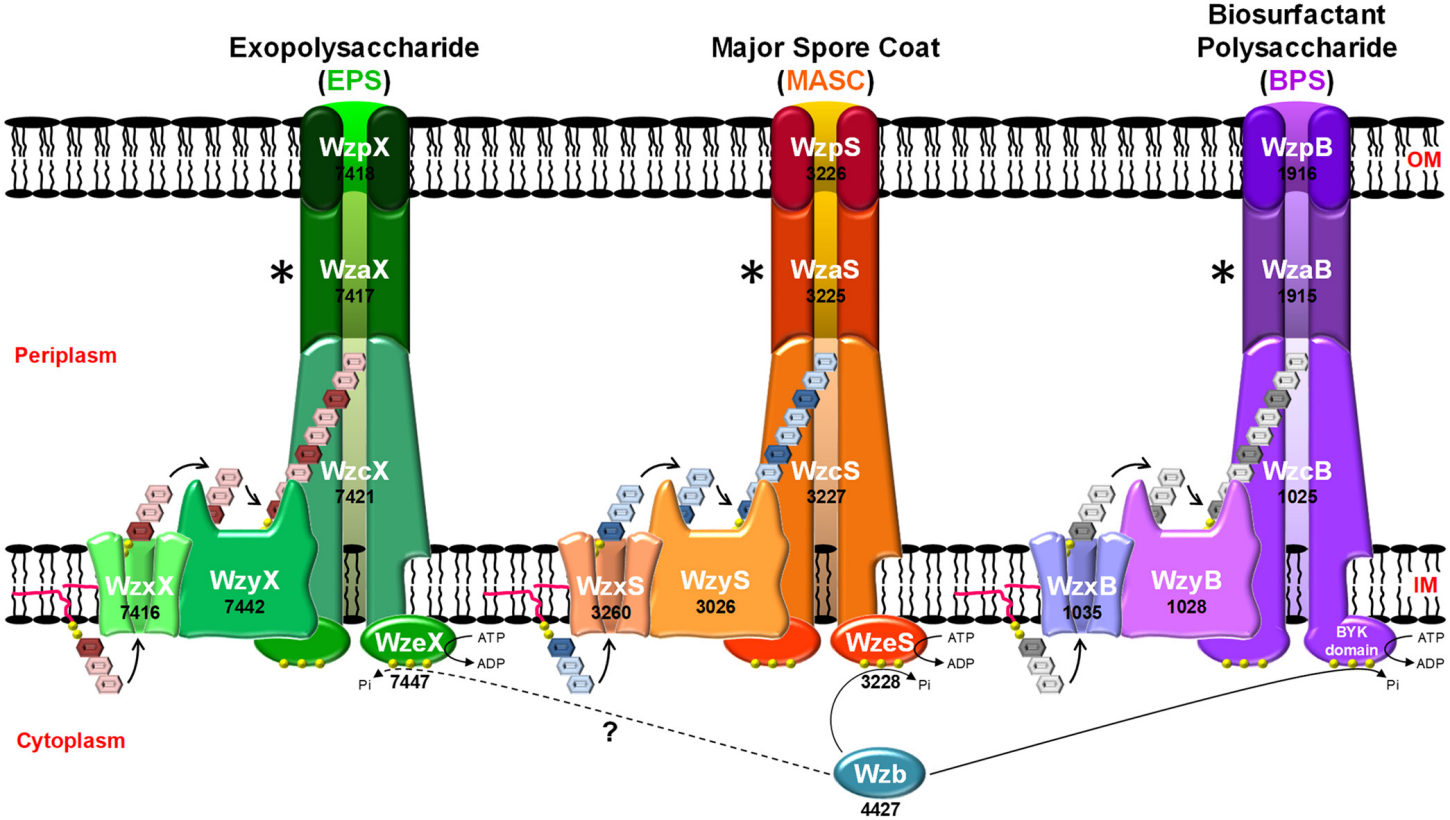
Wzx/Wzy-dependent polysaccharide assembly and secretion pathways in M. xanthus. In these schematics, the WzaX/S/B proteins are depicted in a linking capacity between the apex of the WzcX/S/B PCP periplasmic domains and the periplasmic opening of the integral OM WzpX/S/B β-barrel porins identified here. However, (i) the exposure of the EPS/MASC/BPS polymers to the periplasmic space as each transits between the IM and OM components of each system and (ii) the exact role(s) of the WzaX/S/B proteins in M. xanthus polymer translocation (Fig. S6), remain open questions for each pathway. To denote these uncertainties, this stage of the transport cycle is marked with an asterisk (*).

Ultimately, the findings detailed in this investigation support a model for polysaccharide export in systems lacking integral OM OPX proteins (either Wzx/Wzy- or ABC transporter-dependent) in which the final polymer must pass through an integral OM β-barrel porin for efficient secretion to the cell exterior. For these reasons, as well as the long-established naming convention for polysaccharide assembly-and-secretion proteins in bacteria (60), we propose the designation Wzp (i.e., Wz porin) for the newly identified component of these secretion systems (Fig. 8).

## DISCUSSION

Knowledge of the terminal component through which secreted polysaccharides exit a bacterial cell is crucial for the development of targeted antimicrobial agents that could be used to inhibit this process (61). Here, we have provided evidence that Class 1 and Class 2 OPX proteins have OM-spanning capacities that are absent in Class 3 OPX proteins. Instead, as demonstrated by M. xanthus WzaX/S/B, these Class 3 OPX proteins are genomically and functionally paired with a complementary integral OM-spanning β-barrel porin similar to that required for PNAG export in synthase-dependent pathways.

Class-1 Wza*_Ec_* is the most extensively characterized OPX protein with respect to structure-function relationships. Its OM orientation was established using cell-surface FLAG-epitope immunolabelling of a variant with a C-terminal FLAG tag (35). Similar introduction of a C-terminal His_6_ tag resulted in a partially functional Wza*_Ec_*-His_6_ construct able to restore K30 Group 1 CPS biosynthesis to ~20% of the level restored by an untagged Wza*_Ec_* construct (62). Various C-terminal α-helix truncations also abolished Wza*_Ec_* function (63), indicating that the Wza*_Ec_* OM-spanning domain is functionally sensitive to structural perturbation. Purified Wza*_Ec_*-His_6_ was able to form channel-like 2D octameric ring crystal arrays in lipid bilayers (64), later confirmed by the X-ray crystal structure (35). Using photo-cross-linkable synthetic amino acid *p*-benzoyl-l-phenylalanine (*p*Bpa) at various Wza*_Ec_* sites, Nickerson and colleagues demonstrated that K30 CPS polymers could become trapped in the translocon lumen (36), confirming polysaccharide secretion through Wza*_Ec_*. *p*Bpa substitutions at certain sites within the proximal SLBB domain (Fig. 1A) were able to maintain translocon functionality as well as form polymer cross-links, whereas substitutions within the Poly_export domain rendered Wza*_Ec_* nonfunctional. Conversely, *p*Bpa-substituted positions in the apical SLBB domain, and more importantly, at the kink in the OM-traversing Wza_C domain, maintained Wza*_Ec_* functionality but were unable to form detectable intermolecular cross-links (36). Thus, polymer transit through the Wza_C-domain pore was not demonstrated via this technique. However, purified Wza*_Ec_* in lipid bilayers formed electro-conductive channels, with site-specific amino acid substitutions confirming that ions flow via the Wza_C-domain pore (61). Synthetic peptides corresponding to the native Wza*_Ec_* OM-spanning α-helix were also able to spontaneously insert into such bilayers but formed unstable pores; however, modification of the native Wza_C-domain sequence through consensus generation (based on 94 closely related sequences) yielded an optimized peptide (Fig. 2B) that could spontaneously insert into bilayers and form stable pores (65). Given the primary structure diversity among OM-spanning α-helices uncovered here for both Class 1 and Class 2B OPX proteins (Fig. 2B, 3D), additional optimization of synthetic peptide sequences should be possible to further improve spontaneous membrane insertion, self-assembly, and α-barrel pore stability.

Class 2B OPX protein KpsD*_Ec_*, essential for Group 2 CPS export, was shown to reside in the periplasm when expressed in isolation (66). However, when expressed along with its cognate IM-localized PCP (KpsE*_Ec_*), KpsD*_Ec_* was detected in IM and OM fractions (67). OM-localized KpsD*_Ec_* is multimeric, whereas IM-localized KpsD*_Ec_* is monomeric, indicating KpsD*_Ec_* quaternary structure formation at the site of polysaccharide egress from the cell (32). Immunolabelling of E. coli using α-KpsD*_Ec_* antiserum detected KpsD*_Ec_* epitopes at the cell surface (38), suggesting that a portion of KpsD*_Ec_* was indeed surface accessible. The structural homology detected here of the extreme KpsD*_Ec_* C terminus to the Wza*_Ec_* OM-spanning domain (Fig. 3) supports the contention that a part of KpsD*_Ec_* can interact with and span the OM, albeit conditionally. The 24-residue KpsD*_Ec_* C-terminal α-helix surpasses the 20-residue threshold required to span the hydrophobic core of a membrane bilayer for α-helical integral membrane domains (68). Analogous to Wza*_Ec_*, C-terminal KpsD*_Ec_* truncation by 11 residues abrogated protein function (69). A potential OM-spanning α-helix is a conserved property of the Class 2B OPX proteins identified here. Despite cell surface KpsD*_Ec_* detection with α-KpsD*_Ec_* antibodies, α-His-tag antibodies could not label the surface of cells expressing KpsD*_Ec_*-His_6_ (38). The highly cationic nature of the His_6_ tag may have impeded its translocation across the hydrophobic OM, thus inhibiting immunodetection. Given that KpsD*_Ec_* by itself does not intrinsically associate with the OM (66), this may suggest that the protein can become directly inserted into the OM. Thus, KpsD*_Ec_* could indeed function as the terminal Group 2 CPS secretion component.

Historically, integral OM β-barrel porins had been implicated in Group 2 CPS secretion (70, 71), but such a model has fallen out of favor, as no β-barrels have been detected in Kps clusters, consistent with KpsD*_Ec_*-like Class-2B OPX proteins able to traverse the OM. However, this scenario may not be absolute, as numerous β-barrels encoded near Class 2A/B (as well as Class 1 and Class 3) OPX genes were uncovered here, suggesting that integral OM porins may play an important role in diverse non-synthase-dependent secretion systems. Importantly, Group 4 CPS protein GfcC (together with lipoprotein GfcB) strongly interacts with the β-barrel porin GfcD (72); by extension, Class 2 OPX proteins with integrated C-terminal GfcC modules may indeed interact with partner β-barrel porins. Though not all Class 2 OPX proteins identified herein were matched with a nearby β-barrel, our synteny analysis was limited to ±10 genes from each OPX gene and would not have captured candidate porins elsewhere in genomes; as a case in point, the M. xanthus EPS/MASC/BPS clusters contain >18 kbp/>223 kbp/>1 Mbp insertions that separate constituent members of the same cluster (3). Moreover, as our synteny analyses were limited to 9 query sequences with known polysaccharide associations (8 with PgaA_βb_ homology), this does not preclude the presence of other putative β-barrels (e.g., DUF6029) near unmatched OPX proteins. However, in the absence of specific search templates for confirmed polysaccharide-associated proteins, such analyses were beyond the scope of this investigation.

Identification of Class 3 “OPX” proteins (an obvious misnomer) in Gram-positive bacteria points to a broadly conserved periplasmic function, likely through interfacing with PCP proteins (see below). However, as peptidoglycan-spanning polysaccharide-export channels have yet to be identified in Gram-positive bacteria, any role for OPX proteins with regard to interactions with an unknown secretion pore are unfounded. In M. xanthus, WzaX/S/B Class 3 OPX protein deficiency does not lead to periplasmic accumulation of polymeric material (2), suggesting that Wzx/Wzy-dependent EPS/MASC/BPS assembly does not indiscriminately continue in these mutant backgrounds (similar to Group-1 CPS shutdown in Δ*wza_Ec_* E. coli cells [62]). Such material from ABC transporter-dependent synthesis does, however, accumulate periplasmically in KpsD*_Ec_*-deficient cells (69, 71).

For myxobacterial Class 3 OPX proteins, a clear partnership has now been demonstrated with β-barrel porins to mediate trans-OM polysaccharide export. WzpS (MXAN_3226) was already shown to be essential for MASC secretion, but its function was previously unknown (13). Herein, we have shown that the predicted properties of WzpB (MXAN_1916) make it suitable for BPS translocation across the OM. We have also demonstrated WzpX (MXAN_7418) to be the principal trans-OM EPS export conduit. Residual cell surface EPS is present in Δ*wzpX* cells, which may result from other β-barrel porins (e.g., WzpS/B) inefficiently cross-complementing the EPS pathway deficiency; such inefficiency could arise in part from the cationic natures of the WzpS/B barrel interiors not serving as efficient conduits for a more neutral EPS polymer. Alternatively, depending on the proposed secretion model (see below), OPX proteins from different pathways may not be able to efficiently interface with the native β-barrel for a given polymer.

The presence of a Wza_C domain in a Class 1/2B OPX protein is not mutually exclusive to an integral OM β-barrel protein encoded nearby, considering that such barrels were found encoded near genes for all three OPX protein classes in a range of bacteria (Table 1). GfcD is particularly intriguing given the GfcE (formerly YccZ/Wza_22min_) protein also encoded by the Group 4 CPS locus (50). While GfcD contains a PgaA_βb_-like polysaccharide secretion component and attached SLBB-like domain (Fig. S4C), GfcE is a functional Class 1 OPX Wza*_Ec_* paralogue; this was evidenced by partial restoration of K30 CPS production in E. coli Δ*wza_Ec_* via GfcE expression (73). GfcE possesses a complete Wza_C domain and would thus be expected to span the OM akin to Wza*_Ec_*. The GfcE Wza_C domain may be required to properly interact/organize around the GfcD_Cterβb_ polysaccharide secretion module. Furthermore, the presence of a putative FapF amyloid secretion β-barrel (74) fused to the same polypeptide as that of a PgaA_βb_-like module typically associated with polysaccharide secretion raises an interesting possibility. Amyloid proteins are frequently secreted by bacteria in order to stabilize biofilm matrices composed largely of secreted polysaccharides (75). Thus, in Group 4 capsules, secretion of amyloidogenic polypeptides (via the GfcD FapF-like N-terminal module) could help to stabilize the polysaccharide in the capsule structure and/or anchor the CPS to the cell surface.

**FIG 9:**
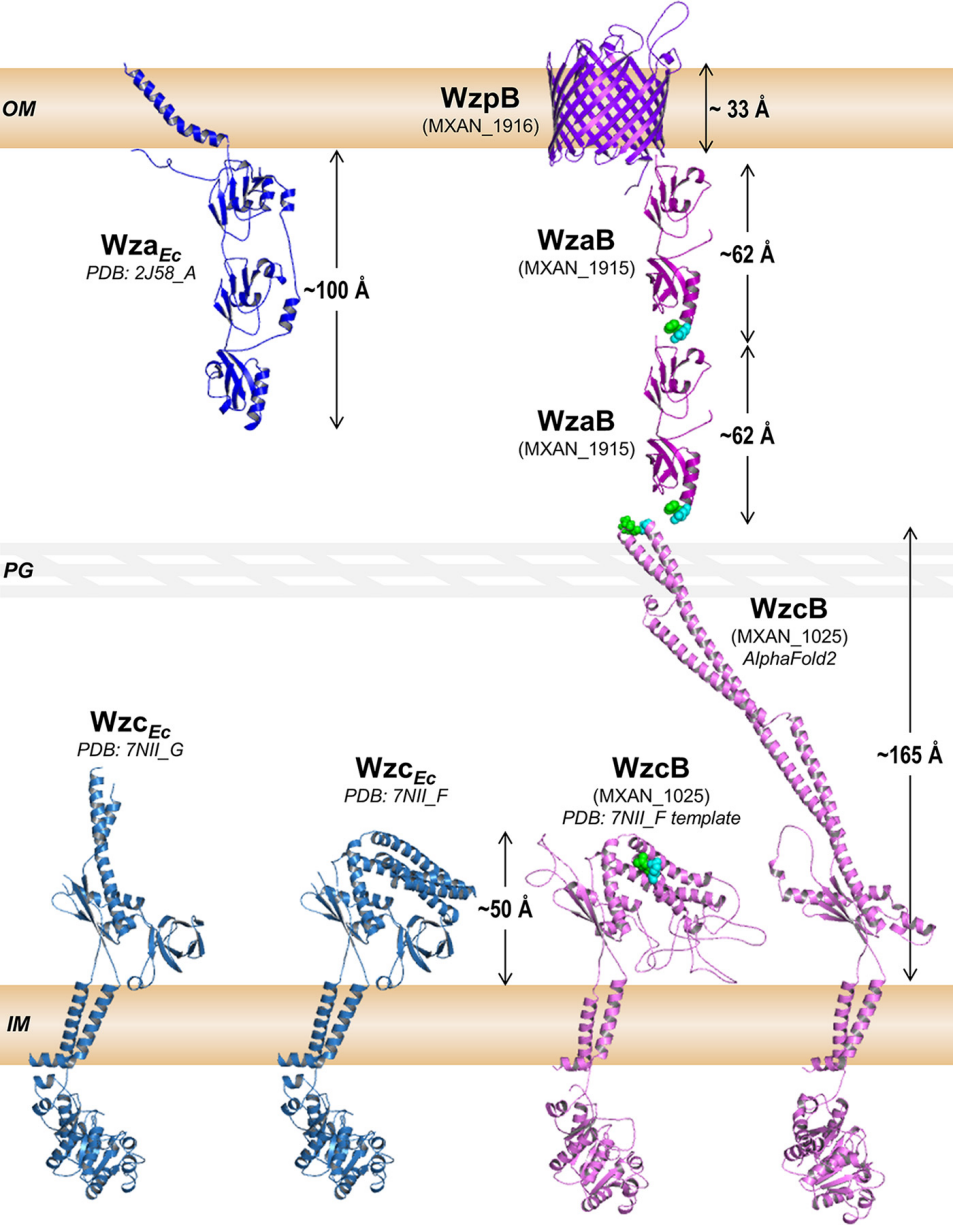
Structural schematic of polymer translocation across the periplasm in M. xanthus. Components from the BPS secretion pathway are used as representative proteins for those in the EPS and MASC pathways as well. All proteins, spaces, and distances are depicted at the same relative scale across a representative 327-Å periplasmic space in an M. xanthus cell. X-ray crystal structures for Wza*_Ec_* (chain A) and Wzc*_Ec_* (chains F and G) are provided as per the PDB files 2J58 (35) and 7NII (25) (respectively) for size references. Structure models for WzaB (Fig. 1B) and WzpB (Fig. 6A) were already generated in this investigation. Models for the PCP protein WzcB were generated using either AlphaFold (resulting in an extended conformation), or MODELLER (specifically against the 7NII_F template, resulting in a compact conformation). High-confidence coevolving amino acids between WzcB and WzaB are highlighted with green (86% probability) and cyan (81% probability) spheres.

**FIG 10:**
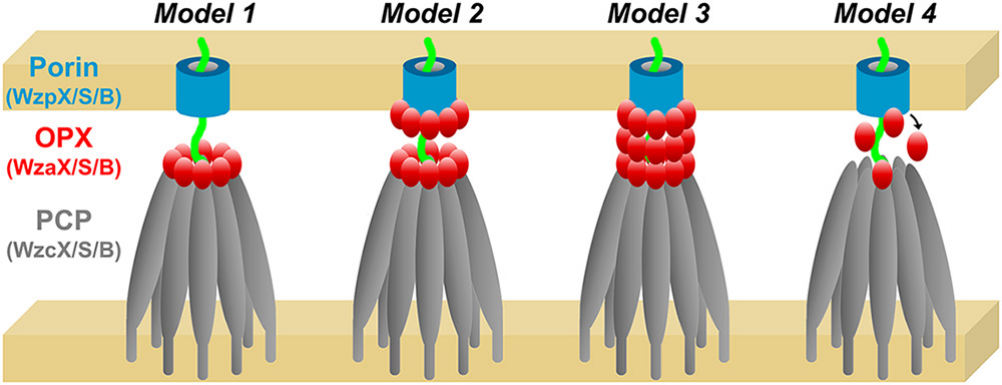
Potential models for polysaccharide export involving Class 3 OPX proteins and β-barrel porins in M. xanthus. Model 1: OPX oligomers interact with PCP oligomers in the periplasm. As the polysaccharide exits the OPX-PCP structure, there is no subsequent targeting mechanism to direct the polymer to its cognate β-barrel porin. Model 2: OPX oligomers interact with PCP oligomers and separately with their cognate β-barrel porin. As the polysaccharide exits the OPX-PCP structure, porin-associated OPX proteins recruit their specific polymer to its cognate β-barrel translocon. Model 3: Contiguous OPX oligomers interact with PCP oligomers, with the former spanning the periplasmic space to reach their cognate β-barrel porin. Model 4: OPX proteins interact with PCP proteins, and as the growing polymer exits the PCP structure, OPX proteins bind the polysaccharide and serve to target it to its cognate β-barrel porin. Once the polymer-bound OPX protein reaches the porin, it can detach and be reused for subsequent cycles of binding PCP proteins followed by polysaccharide engagement.
Model 2: A variation of model 1 in which single-layer periplasmic WzaB oligomers interact with the PCP-octamer apex and the proximal face of the WzpB β-barrel. OPX protein-mediated substrate specificity would thus exist at both ends of the transport process, but again, the polymer could be exposed to the periplasm during transport between OPX protein layers (Fig. 10). Nonetheless, this model provides a solution to the targeting dilemma of the nascent polymer to the proper OM-spanning apparatus. However, this also presumes a constant OM-associated Class 3 OPX-protein presence, as well as specific WzpB–WzaB interactions. The latter is not bolstered by evolutionary-coupling data between WzpB and WzaB (Table S5), but this may be partially due to an insufficient number of barrel homologues with which to fully probe coevolution. However, as OPX proteins were not detected in M. xanthus surface-biotinylated protein, OMV, or OM-fractionated samples (39, 48), experimental evidence for this concept is lacking.
Model 3: A total of 327 Å of periplasmic distance could be accounted for by ~165 Å of WzcB domain height, followed by WzaB oligomers stacked in duplicate (~62 Å × 2 = ~124 Å) or triplicate (~62 Å × 3 = ~186 Å), depending on the oligomer-packing arrangement. Along with the PCP channel, such architecture could form a protected channel from the IM to the OM, precluding periplasmic exposure of the polymer (Fig. 10). This would also abrogate any targeting issues of the polymer to its β-barrel porin. Periplasmic high-molecular-weight M. xanthus OPX protein oligomers would thus be expected, which could coprecipitate with IM and/or OM fractions. Data to support this contention await further experimentation.
Model 4: As the nascent polymer emerges from a WzcB channel, it interacts with WzaB units at the WzcB apex, allowing WzaB to bind the polymer and detach from WzcB. As polymer elongation occurs, more WzaB units bind along the polymer. Polymer-bound WzaB would thus serve as a type of targeting chaperone to preferentially direct the periplasmic polymer to its cognate β-barrel porin. Once a given WzaB has reached the porin, it would disengage from the polymer to undergo subsequent rounds of binding to the nascent polymer (Fig. 10). This would afford a degree of protection to the translocating polymer against the periplasm and provide a polymer-targeting mechanism to the specific β-barrel porins needed for transport across the OM. This would also account for the nondetection of WzaX/S/B in surface-biotinylated-protein or OMV proteomic screens (39); i.e., that WzaX/S/B OM association (via WzpX/S/B interaction) is more transient in nature.

For Class 3 OPX proteins (e.g., WzaX/S/B), the small size and lack of OM-spanning domains present a dilemma for polysaccharide transit across the periplasm when analyzed at scale (Fig. 9). To illustrate this issue, we have used various components of the *M. xanthus* BPS pathway as stand-ins for the following descriptions. First, the M. xanthus periplasm is ~327 Å thick (Fig. 5A and B), while the periplasmic domain of the BPS pathway PCP (WzcB) may extend up to ~165 Å from the IM. Coupled with an ~62-Å maximum height for one WzaB, this only occupies ~227 Å of periplasmic distance, leaving ~100 Å unaccounted for (Fig. 9) compared to standard polysaccharide-secretion models (Fig. 1A). High-confidence coevolving residues between WzcB and WzaB localize to the PCP apex and OPX-protein Poly_export base (respectively) (Fig. 9, green and cyan highlighted residues; Table S5), heavily implying a conserved WzcB–WzaB interaction interface. Using BPS-pathway components as examples (Fig. 8), several potential models for trans-envelope transit can thus be proposed:

Model 1: As the polymer exits the periplasmic WzcB cavity (following WzyB-mediated polymerization), it passes through a WzcB-associated single-layer WzaB oligomer. The polymer would then have to locate its cognate integral OM WzpB β-barrel porin to exit the cell. This presumes the polymer is periplasmically exposed for a substantial portion of its transit between the IM and OM (Fig. 10). In this model, polymer export might still be possible in the absence of the cognate OPX (as long as the absence of the latter does not impact polymer assembly), but this is not the case in M. xanthus.

Periplasmic exposure of a sugar polymer during transit between the IM and OM is common in alginate, cellulose, and PNAG synthase-dependent systems (31). Recently, Group 2 CPS was also suggested to be periplasmically exposed at some point during trans-envelope transport (76). There, periplasmic enzymes can access the transiting polymer and introduce modifications, including sugar epimerization and/or (de)acetylation. This may be applicable for M. xanthus BPS, for which the secreted form displays random acetylation (3). In synthase-dependent pathways, tetratricopeptide-repeat domains (either standalone or attached) extend into the periplasm from the β-barrel porin (Fig. S5) and are proposed to interact with chain-modifying enzymes (31). Of note, numerous OPX proteins were found to be encoded near full-length PgaA and BcsC β-barrel homologues complete with the respective tetratricopeptide-repeat architectures (Table 1). In these systems, questions arise as to the functional relationships between periplasmic OPX and integral OM β-barrel proteins, with tetratricopeptide-repeat domains potentially occluding OPX protein access to the periplasmic face of the porin.

Ultimately, our study reveals OPX protein complexities in diverse organisms that differ from the well-studied Wza*_Ec_* protein, which will further our mechanistic understanding of bacterial polysaccharide export. Detailed biochemical and biophysical characterizations of protein complex formation will be required to tease apart the interactions between PCP, OPX, and newly identified β-barrel constituents of polysaccharide assembly and secretion pathways. Updated genomic, structural, and functional knowledge of the terminal polysaccharide secretion step will thus enable researchers to selectively develop novel antimicrobial compounds targeted to blocking bacterial polymer secretion from the outside, thus bypassing any requirements for access to the cell interior to compromise bacterial cell viability.

### Note added in proof.

During the review process for this manuscript, Schwabe and colleagues posted a preprint (77) showing that (i) the M. xanthus OPX proteins WzaX, WzaS, and WzaB are truncated compared to Wza*_Ec_* (without OM-spanning α-helices), (ii) unidentified truncated OPX proteins are found across a panel of bacterial genomes surveyed, and (iii) WzaX/S/B are genomically paired with adjacently encoded 18-stranded β-barrel proteins. (iv) Moreover, these β-barrel proteins are consistent with integral OM porins for each of the three major M. xanthus polysaccharide secretion pathways. Also, (v) targeted gene deletion of the exopolysaccharide (EPS)-pathway β-barrel porin WzpX results in a qualitative abrogation in EPS secretion; however, the Δ*wzpX* strain still displays residual T4P-dependent motility compared to an EPS-deficient strain. These subsequent findings provide valuable independent verification for a subset of the results and conclusions we have detailed in our current investigation.

## MATERIALS AND METHODS

### Protein structure analysis and modeling.

The WzaX/S/B tertiary structure was modeled against the Wza*_Ec_* template (PDB: 2J58) using MODELLER. MXAN_7418/3226/1918 structure models were computed using deep learning and artificial intelligence via AlphaFold (55). Multiple-sequence-alignment entries were generated using 101/251/606 unique sequences for MXAN_7418/3226/1916, with the program run for 5 independent prediction models, leading to convergence after 3 recycling iterations. For GfcD and YjbH, AlphaFold-generated structures were mined from the P75882 and P32689 UniProt entries, respectively. HOLLOW (78) was used to generate internal MXAN_7418/3226/1916 volume casts and overlay the solvent-accessible electrostatic potential contributed by amino acids in contact with the internal volume, as calculated using PDB2PQR and APBS (Propka pH 7.0; Swanson force field, ±5 kT/e). All protein structures were visualized and rendered in PyMol. Evolutionarily coupled amino acids within the same protein were analyzed using RaptorX-Contact (79), while those between two proteins were determined using RaptorX-ComplexContact (80). Protein contact maps were displayed using GraphPad software.

### OPX protein identification.

Three data sets (61 order *Myxococcales* genomes [MYXO], 3,662 reference bacterial genomes [REP; extracted from the Prokaryotes.txt file downloaded 7 December 2021 from https://ftp.ncbi.nlm.nih.gov/genomes/GENOME_REPORTS/prokaryotes.txt], and nonredundant NCBI database [NR] [371,327,556 proteins at 100% identified as of 10 June 2021]) were downloaded from NCBI. Pfam domains PF02563 (Poly_export), PF10531 (SLBB), PF18412 (Wza_C), and PF06251 (Caps_synth_GfcC; referred to here as GfcC) were extracted from the Pfam-A v34.0 database (81) (downloaded 24 March 2021), and a reduced combined profile database was created. These domains were identified by scanning the three databases for the Pfam entries using offline hmmscan (82) (E-value cutoff of 1 × 10^−5^), followed by parsing via hmmscan-parser.sh, and arranging in the form of protein architecture using in-house scripts. Based on domains identified per protein per data set, three primary clusters were identified. All primary-cluster proteins were subjected to fold-recognition analysis using HHpred (83) against the database of two proteins (PDB: 2J58 [Wza*_Ec_*] and PDB: 3P42 [GfcC]; extracted from PDBmmCIF70, downloaded on 19 November 2021) using the parameters -p 5 -Z 500 -loc -z 1 -b 1 -B 500 -all -id 35 -ssm 2 -sc 1 -seq 1 -dbstrlen 10000 -norealign -maxres 32000 parameters. HHpred raw data were parsed using in-house scripts to generate the architecture of each protein in terms of nonoverlapping homologous regions to 2J58 and 3P42. SignalP v6.0 (84) and the TMHMM server v2.0 (85) were used to identify signal peptides and membrane topology, respectively.

Using HHpred, we also identified the secondary structure-based homology of the Wza_C segment (aa 326 to 359; 34-aa length) in 2J58. Homology with at least 10/34 amino acids of the Wza_C segment (aa 326 to 359) in 2J58 was considered a true Wza_C segment in the respective protein. Proteins with both 2J58- and 3P42-homologous nonoverlapping regions were classified as Class 2. Proteins with only 2J58-homologous regions along with a Wza_C segment were classified as Class 1. Other proteins with only 2J58-homologous regions and no Wza_C segment were classified as Class 3. WebLogo v2.8 (86) was used to generate sequence logos for Wza_C segments in Class 1 and Class 2B OPX proteins.

### Synteny analysis of β-barrel query proteins with identified OPX proteins.

β-barrel homologues encoded in the vicinity (±10 genes) of OPX genes were identified in the MYXO and REP data sets using BLASTp and hmmscan by scanning against protein sequences from M. xanthus DZ2 (MXAN_7418 [aa 24 to 415], MXAN_3226 [aa 23 to 381], and MXAN_1916 [aa 26 to 421]), E. coli K12 (PgaA [aa 511 to 807], GfcD [aa 425 to 698], YjbH [aa 423 to 698], BcsC [aa 785 to 1157], and Wzi [aa 92 to 479]), and Pseudomonas aeruginosa PAO1 (AlgE [aa 33 to 490]). Detected homologues were aligned against the truncated and full-length sequences for each query protein using Clustal Omega to probe for the lone presence of a putative β-barrel polysaccharide secretion porin domain versus its presence as part of a multidomain polypeptide comparable to the native sequence. Hits resembling truncated versions of various β-barrel queries were individually profiled via fold recognition using the online HHpred toolkit (87). Depicted sequence alignments were displayed in GeneDoc with residues colored according to conservation score (out of 10) as indicated in Jalview (88).

### Phylogenetic analysis of OPX proteins.

The PF02563 (Poly_export) domain sequence of OPX proteins was used as a phylogenetic marker. The locations of all identified Poly_export domains were first extracted, after which, extracted sequences were aligned using MUSCLE (with 10 iterations). The resultant alignment was analyzed via FastTree v2.1.10 to generate a maximum likelihood tree of OPX proteins. Finally, tree visualization and mapping of the OPX classification, presence of nearby β-barrels, taxonomy, and length of each branch were performed via the iTOL web server v6.4.3 (89).

### Bacterial membrane modeling and intermembrane distance measurements.

The desired prokaryotic tomograms were downloaded from the Caltech Electron Tomography Database (https://etdb.caltech.edu/) (47). A total of 40 species were analyzed, each via 3 separate tomograms. Tomogram inspection and modeling were performed using the IMOD software package (90). Tomograms were first oriented in 3D using the IMOD slicer window to identify the central slice through each bacterium. To enhance contrast, 5 layers of voxels were averaged around the section of interest. Model points were then placed along corresponding regions of the OM and IM for a total distance of ~100 nm. A custom Python script was subsequently used to calculate the intermembrane distance every 0.1 nm along the modeled stretch of membranes. The script is available to readers upon request. GraphPad software was used to prepare plots and carry out correlation analyses.

### Bacterial cell culture.

Wild-type M. xanthus DZ2 (91) and isogenic mutants (Table 2) were grown and maintained at 32°C on Casitone-yeast extract (CYE; 1% w/v casitone, 0.5% w/v yeast extract, 10 mM MOPS [pH 7.5], 4 mM MgSO_4_, 1.5% w/v agar) plates or in CYE liquid medium at 32°C on a rotary shaker at 220 rpm.

**TABLE 2:** Strains and plasmids used in this study

Strain or Plasmid	Genotype/description	Source or reference
Strains		
DZ2	Myxococcus xanthus (wild type)	91
TM389	DZ2 Δ*pilA* (Δ*mxan_5783*)	95
TM469	DZ2 Δ*wzaX* (i.e., Δ*mxan_7417/epsY*)	95
SI93	DZ2 Δ*wzpX* (i.e., Δ*mxan_7418/epsX*)	This study
SI94	DZ2 Δ*wzpX attP*::pSWU19, Km^R^	This study
SI95	DZ2 Δ*wzpX attP*::pSWU19-*wzpX* (P*_pilA_ wzpX*), Km^R^	This study
DH5α	Escherichia coli (cloning strain); F^−^ φ80*lac*ZΔM15 Δ(*lac*ZYA-*arg*F)U169 *rec*A1 *end*A1 *hsd*R17(rκ^−^, mκ^+^) *pho*A *sup*E44 λ^−^*thi*-1 *gyr*A96 *rel*A1	Laboratory stock
Plasmids		
pBJ114	pUC118 derivative, containing Km^R^ and *galK* (from pKG2); plasmid used for constructing gene replacements	93
pBJ114-Δ*wzpX*	pBJ114 containing a recombination construct to delete nucleotides 7–1229 from *mxan_7418*	This study
pSWU19	Km^R^, for single-copy integration at Mx8 *attB* phage-attachment site	94
pSWU19-*mxan_7418* (*wzpX*)	pSWU19 containing a P*_pilA_ wzpX* construct to complement a MXAN_7418 deficiency, Km^R^	This study

### Mutant construction.

As previously described (3), to generate an M. xanthus deletion mutant strain, 528 bp upstream and 583 bp downstream of the target gene were amplified via PCR and fused via gene splicing by overlap extension (SOEing) (92) and cloned into pBJ114 (93) between the EcoRI and HindIII restriction sites using T4 DNA ligase. Ligation products were used to transform chemically competent E. coli DH5α via heat shock treatment, with recovery cultures grown in LB liquid medium and plated on LB agar plates containing kanamycin (50 μg/mL), all grown at 37°C. Drug-resistant colonies were amplified, from which plasmids were isolated using a miniprep kit (Promega). Clones confirmed via Sanger sequencing were introduced into M. xanthus DZ2 via electroporation (900 V voltage, 25 μF capacitance, 400 Ω resistance, 2 mm electroporation cuvette), followed by selection on CYE agar containing kanamycin (100 μg/mL) and then CYE agar containing galactose (2.5%) to obtain the final deletion strain, which was verified by flanking PCR and sequencing of the product.

For complementation assays, the *mxan_7418* gene was commercially synthesized with an upstream *pilA* promoter and, then cloned in the pSWU19 plasmid (94), between the HindIII and EcoRI restriction sites, to yield the pSWU19-*mxan_7418* plasmid (GenScript). To insert plasmids at the Mx8 phage-attachment site in the M. xanthus genome, either empty pSWU19 or pSWU19-*mxan_7418* was introduced via electroporation, followed by selection of kanamycin-resistant clones, both as described above.

### Trypan blue dye retention.

Retention of trypan blue was carried out as previously described (3). Cells from overnight cultures were resuspended in Tris-phosphate-magnesium (TPM; 10 mM Tris-HCl [pH 7.6], 1 mM KH_2_PO_4_, 8 mM MgSO_4_) buffer (optical density at 600 nm [OD_600_], 1.0), 900 μL of which was then mixed with 100 μL trypan blue solution (100 μg/mL stock). Samples were covered and incubated (1 h) on a rocker platform at room temperature and sedimented (16,000 × *g*, 5 min), after which the top 900 μL of clarified supernatant was transferred to a spectrophotometer cuvette. A cell-free TPM plus trypan blue sample was used to blank the spectrophotometer (585 nm). Absorbance at 585 nm was determined for each sample and then normalized to that for the WT of each biological replicate. Subzero final values are due to trace amounts of cell debris detected at 585 nm in individual samples in which no binding of trypan blue occurred.

### Phenotypic analyses.

Cells from exponentially growing cultures were harvested and then resuspended in TPM buffer (final concentration, OD_600_ 5.0). For T4P-dependent swarm expansion, cell suspension (5 μL) was spotted onto CYE 0.5% agar. Plates were incubated at 32°C (72 h) and then imaged with an Olympus SZX16 stereoscope with a UC90 4K camera, with images captured using the 0.5× objective at 1× zoom (linear color, dark-field illumination).

### Auto-aggregation testing.

The protocol followed has been previously detailed (2). Specific culture volumes were aspirated and then sedimented via microfuge (4,000 × *g*, 5 min) so that pellet resuspension in 1 mL CYE broth would yield a final OD_600_ of 1.0. Samples were transferred to a spectrophotometer cuvette, with resuspensions strongly aspirated and ejected (10 s) and then immediately read for OD_600_ (*t *= 0) via spectrophotometer. OD_600_ time course readings were taken every 10 min for 100 min, with cuvettes left covered and undisturbed on the benchtop in between readings. All OD_600_ readings were normalized to that determined at *t *= 0 for each sample.
