# Supplementary material for: Bacterial Outer Membrane Polysaccharide Export (OPX) Proteins Occupy Three Structural Classes with Selective β-Barrel Porin Requirements for Polymer Secretion
Source: Microbiol Spectr. 2022 Oct 6;10(5):e01290-22. doi: 10.1128/spectrum.01290-22 (PMC9603273; doi:10.1128/spectrum.01290-22)
Supplement: Supplemental file 1 — Figures S1-S5, Table S1-S5 captions. Download spectrum.01290-22-s0001.pdf, PDF file, 2.2 MB [file spectrum.01290-22-s0001.pdf]

SUPPLEMENTAL MATERIAL

Bacterial outer-membrane polysaccharide export (OPX) proteins occupy three structural classes with selective β-barrel porin requirements for polymer secretion

Running Title: β-barrel porin requirements for OPX-mediated polysaccharide secretion

Fares Saïdi[1,2†], Utkarsha Mahanta[3†], Adyasha Panda[3], Ahmad A. Kezzo[1,2], Nicolas Y. Jolivet[1,2], Razieh Bitazar[1,2], Gavin John[4], Matthew Martinez[5], Abdelkader Mellouk[1,2], Charles Calmettes[1,2], Yi-Wei Chang[5], Gaurav Sharma[3,6*], Salim T. Islam[1,2*]

[1] Institut National de la Recherche Scientifique (INRS), Centre Armand-Frappier Santé Biotechnologie, Université du Québec, Institut Pasteur International Network, Laval, QC, Canada

[2] PROTEO, the Quebec Network for Research on Protein Function, Engineering, and Applications, Université Laval, Québec, QC, Canada

[3] Institute of Bioinformatics and Applied Biotechnology (IBAB), Bengaluru, Karnataka, India

[4] Department of Pediatrics, Division of Infectious Diseases, Children's Hospital of Philadelphia, Philadelphia, PA, U.S.A.

[5] Department of Biochemistry and Biophysics, Perelman School of Medicine, University of Pennsylvania, Philadelphia, PA, U.S.A.

[6] Department of Biotechnology, Indian Institute of Technology Hyderabad, Sangareddy, Telangana, India (present address)

[†]co-1st authors

*corresponding authors:

Salim T. Islam
salim.islam@inrs.ca
ORCID ID: 0000-0001-6853-8446

Gaurav Sharma
gaurav.amit30@gmail.com
ORCID ID: 0000-0002-2861-7446

Figure S1. Structural analysis of MXAN_7418 (WzpX). (A) Evolutionarily-coupled amino acids within the MXAN_7418 primary structure (determined via RaptorX). **(B)** Fold-recognition analysis of MXAN_7418 (via HHpred) revealing N-terminal structural homology with two β-strands from FhuA (PDB: 4CU4) (1), with the remainder of the protein displaying structural homology to $PgaA_{\beta b}$ (PDB: 4Y25) (2). FhuA and $PgaA_{\beta b}$ β-strand (*dark blue arrows*) structure is depicted as per the respective PDB entries. MXAN_7418 predicted β-strand (*light blue arrows*) secondary structure is indicated as per PSIPRED analysis. Aligned residues have been coloured according to Jalview conservation score (out of 10). *Maroon*, 10; *red*, 9; *orange*, 8; *yellow*, 7; *pink*, 6. Scores of 5 or less have been omitted to improve clarity of the figure.

FIGURE S1

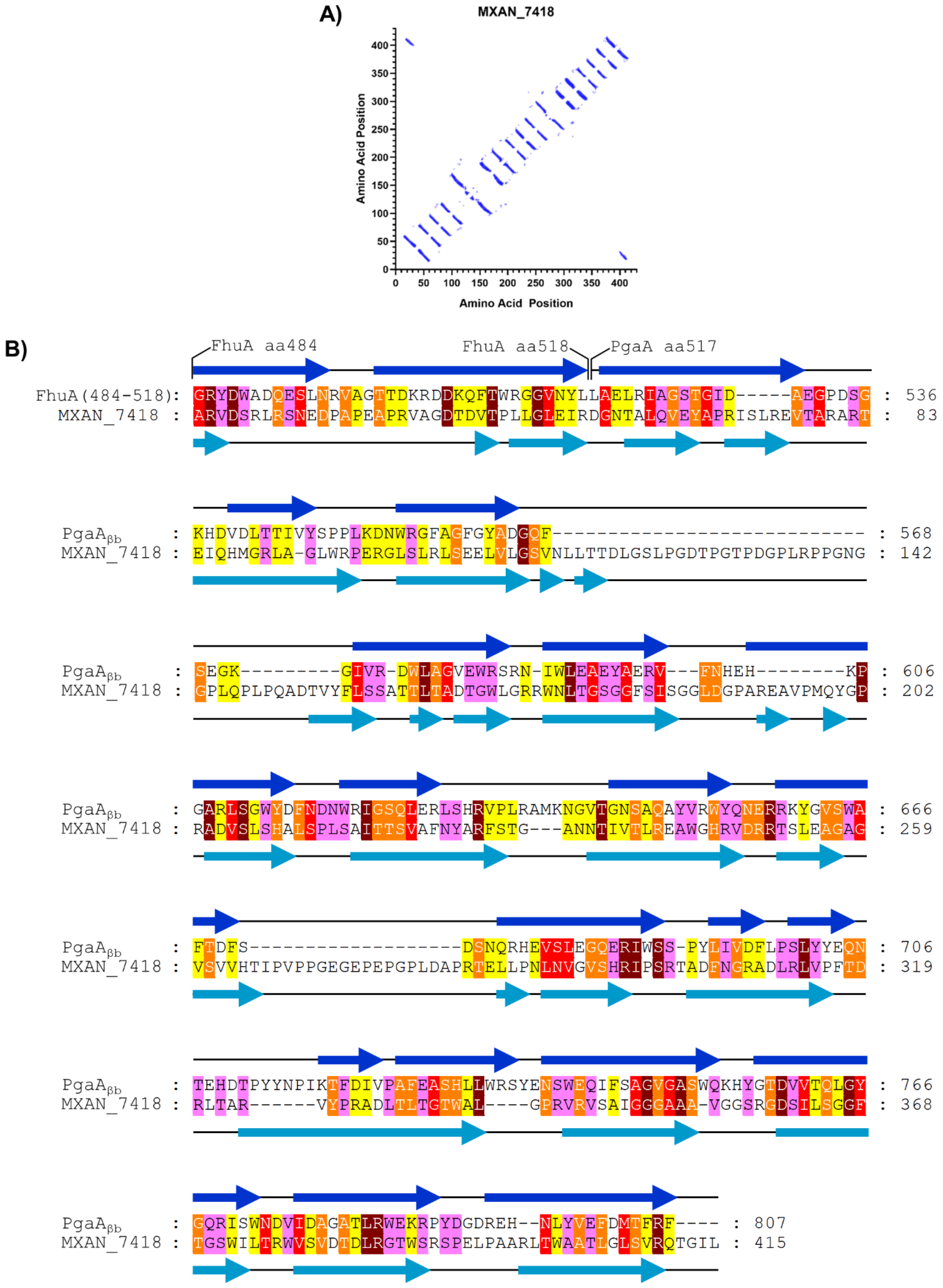
A)
MXAN_7418
Amino Acid Position
0
50
100
150
200
250
300
350
400
Amino Acid Position
B)
FhuA aa484
FhuA aa518
PgaA aa517
FhuA(484-518): GRYDWADQESLNRVAGTTDKRDDKQFTWRGGVNYLLAELRIAGSTGID-----AEGPDSG : 536
MXAN_7418 : ARVDSRLRSNEDPAPEAPRVAGDTDVTPLLGLEIRDGNTALQVEYAPRISLREVTARART : 83
PgaAββb : KHDVDLTTIVYSPPLKDNWRGFAGFGYADGQF---------------------------- : 568
MXAN_7418 : EIQHMGRLA-GLWRPERGLSLRLSEELVLGSVNLLTTDLGSLPGDTPGTPDGPLRPPGNG : 142
PgaAββb : SEGK---------GIVR-DWLAGVEWRSRN-IWLEAEYAERV---FNHEH--------KP : 606
MXAN_7418 : GPLQPLPQADTVYFLSSATTLTADTGWLGRRWNLTGSGGFSISGGLDGPAREAVPMQYGP : 202
PgaAββb : GARLSGWYDFNDNWRIGSQLERLSHRVPLRAMKNGVTGNSAQAYVRWYQNERRKYGVSWA : 666
MXAN_7418 : RADVSLSHALSPLSAITTSVAFNYARFSTG---ANNTIVTLREAWGHRVDRRTSLEAGAG : 259
PgaAββb : FTDFS-------------------DSNQRHEVSLEGQERIWSS-PYLIVDFLPSLYYEQN : 706
MXAN_7418 : VSVVHTIPVPPGEGEPEPGPLDAPRTELLPNLNVGVSHRIPSRTADFNGRADLRLVPFTD : 319
PgaAββb : TEHDTPYYNPIKTFDIVPAFEASHLLWRSYENSWEQIFSAGVGASWQKHYGTDVVTQLGY : 766
MXAN_7418 : RLTAR------VYPRADLTLTGTWAL----GPRVRVSAIGGGAAA-VGGSRGDSILSGGF : 368
PgaAββb : GQRISWNDVIDAGATLRWEKRPYDGDREH--NLYVEFDMTFRF---- : 807
MXAN_7418 : TGSWILTRWVSVDTDLRGTWSRSPELPAARLTWAATLGLSVRQTGIL : 415

Figure S2. Structural analysis of MXAN_3226 (WzpS). (A) Evolutionarily-coupled amino acids within the MXAN_3226 primary structure (determined via RaptorX). **(B)** Fold-recognition analysis of MXAN_3226 (via HHpred) revealing N-terminal structural homology with two β-strands from NanC (PDB: 2WJR) (3), with the remainder of the protein displaying structural homology to $PgaA_{\beta b}$ (PDB: 4Y25) (2). NanC and $PgaA_{\beta b}$ β-strand (*dark blue arrows*) structure is depicted as per the respective PDB entries. MXAN_3226 predicted β-strand (*light blue arrows*) secondary structure is indicated as per PSIPRED analysis. Aligned residues have been coloured according to Jalview conservation score (out of 10). *Maroon*, 10; *red*, 9; *orange*, 8; *yellow*, 7; *pink*, 6. Scores of 5 or less have been omitted to improve clarity of the figure.

FIGURE S2

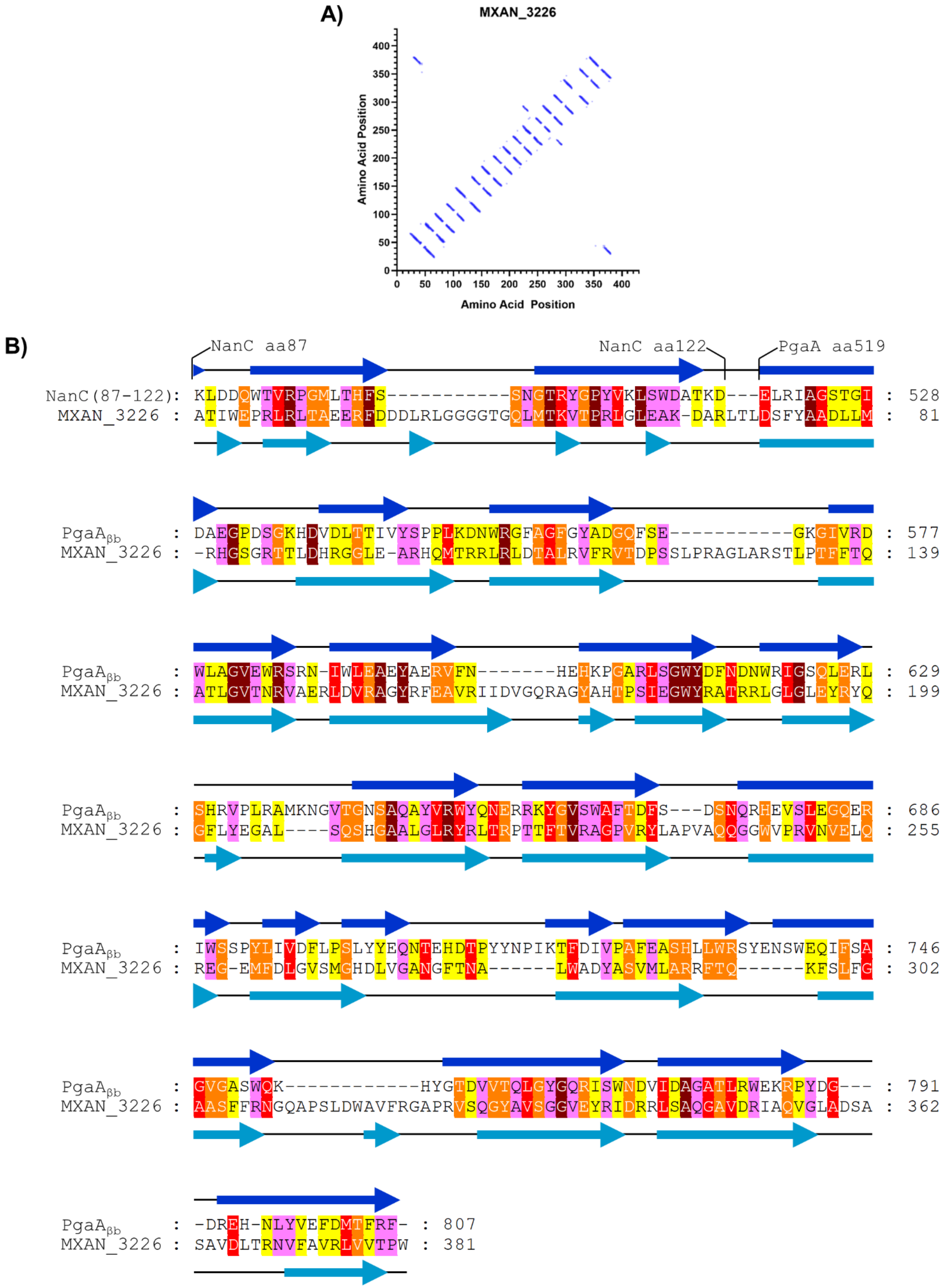
A)
MXAN_3226
Amino Acid Position
Amino Acid Position
0 50 100 150 200 250 300 350 400
B)
NanC aa87
NanC aa122
PgaA aa519
NanC(87-122): KLDDQWTVRPGMLTHFS-----------SNGTRYGPYVKLSWDATKD---ELRIAGSTGI : 528
MXAN_3226 : ATIWEPRLRLTAEERFDDDLRLGGGGTGQLMTKVTPRLGLEAK-DARLTLDSFYAADLLM : 81
PgaAβb : DAEGPDSGKHDVDLTTIVYSPPLKDNWRGFAGFGYADGQFSE-----------GKGIVRD : 577
MXAN_3226 : -RHGSGRTTLDHRGGLE-ARHQMTRRLRLDTALRVFRVTDPSSLPRAGLARSTLPTFFTQ : 139
PgaAβb : WLAGVEWRSRN-IWLEAEYAERVFN-------HEHKPGARLSGWYDFNDNWRIGSQLERL : 629
MXAN_3226 : ATLGVTNRVAERLDVRAGYRFEAVRIIDVGQRAGYAHTPSIEGWYRATRRLGLGLEYRYQ : 199
PgaAβb : SHRVPLRAMKNGVTGNSAQAYVRWYQNERRKYGVSWAFTDFS---DSNQRHEVSLEGQER : 686
MXAN_3226 : GFLYEGAL----SQSHGAALGLRYRLTRPTTFTVRAGPVRYLAPVAQQGGWVPRVNVELQ : 255
PgaAβb : IWSSPYLIVDFLPSLYYEQNTEHDTPYYNPIKTFDIVPAFEASHLLWRSYENSWEQIFSA : 746
MXAN_3226 : REG-EMFDLGVSMGHDLVGANGFTNA------LWADYASVMLARRFTQ------KFSLFG : 302
PgaAβb : GVGASWQK------------HYGTDVVTQLGYGQRISWNDVIDAGATLRWEKRPYDG--- : 791
MXAN_3226 : AASFFRNGQAPSLDWAVFRGAPRVSQGYAVSGGVEYRIDRRLSAQGAVDRIAQVGLADSA : 362
PgaAβb : -DREH-NLYVEFDMTFRF- : 807
MXAN_3226 : SAVDLTRNVFAVRLVVTPW : 381

Figure S3. Structural analysis of MXAN_1916 (WzpB). (A) Evolutionarily-coupled amino acids within the MXAN_1916 primary structure (determined via RaptorX). **(B)** Fold-recognition analysis of MXAN_1916 (via HHpred) revealing N-terminal structural homology with two β-strands from PagL (PDB: 2ERV) (4), with the remainder of the protein displaying structural homology to $PgaA_{\beta b}$ (PDB: 4Y25) (2). PagL and $PgaA_{\beta b}$ β-strand (*dark blue arrows*) structure is depicted as per the respective PDB entries. MXAN_1916 predicted α-helix (*light green cylinders*) and β-strand (*light blue arrows*) secondary structure is indicated as per PSIPRED analysis. Aligned residues have been coloured according to Jalview conservation score (out of 10). *Maroon*, 10; *red*, 9; *orange*, 8; *yellow*, 7; *pink*, 6. Scores of 5 or less have been omitted to improve clarity of the figure.

FIGURE S3

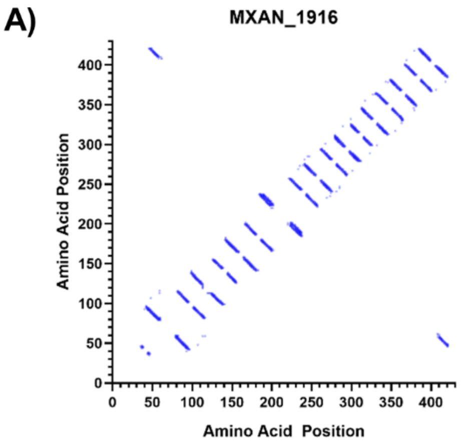
A)
MXAN_1916
Amino Acid Position
Amino Acid Position

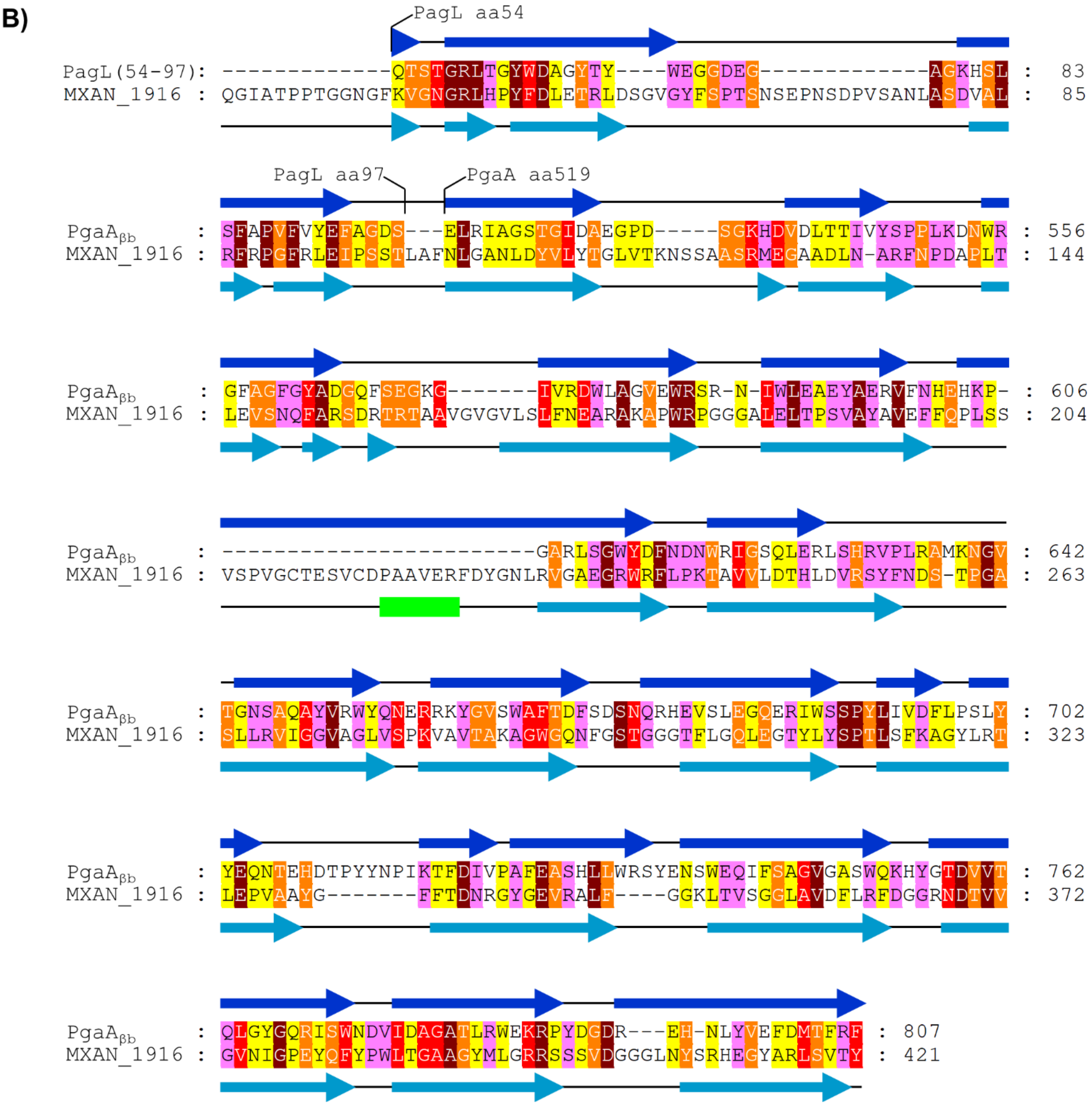
B)
PagL aa54
PagL(54-97): -------------QTSTGRLTGYWDAGYTY----WEGGDEG-------------AGKHSL : 83
MXAN_1916 : QGIATPPTGGNGFKVGNGRLHPYFDLETRLDSGVGYFSPTSNSEPNSDPVSANLASDVAL : 85
PagL aa97
PgaA aa519
PgaAβb : SFAPVFVYEFAGDS---ELRIAGSTGIDAEGPD-----SGKHDVDLTTIVYSPPLKDNWR : 556
MXAN_1916 : RFRPGFRLEIPSSTLAFNLGANLDYVLYTGLVTKNSSAASRMEGAADLN-ARFNPDAPLT : 144
PgaAβb : GFAGFGYADGQFSEGKG-------IVRDWLAGVEWRSR-N-IWLEAEYAERVFNHEHKP- : 606
MXAN_1916 : LEVSNQFARSDRTRTAAVGVGVLSLFNEARAKAPWRPGGGALELTPSVAYAVEFFQPLSS : 204
PgaAβb : ------------------------GARLSGWYDFNDNWRIGSQLERLSHRVPLRAMKNGV : 642
MXAN_1916 : VSPVGCTESVCDPAAVERFDYGNLRVGAEGRWRFLPKTAVVLDTHLDVRSYFNDS-TPGA : 263
PgaAβb : TGNSAQAYVRWYQNERRKYGVSWAFTDFSDSNQRHEVSLEGQERIWSSPYLIVDFLPSLY : 702
MXAN_1916 : SLLRVIGGVAGLVSPKVAVTAKAGWGQNFGSTGGGTFLGQLEGTYLYSPTLSFKAGYLRT : 323
PgaAβb : YEQNTEHDTPYYNPIKTFDIVPAFEASHLLWRSYENSWEQIFSAGVGASWQKHYGTDVVT : 762
MXAN_1916 : LEPVAAYG--------FFTDNRGYGEVRALF----GGKLTVSGGLAVDFLRFDGGRNDTVV : 372
PgaAβb : QLGYGQRISWNDVIDAGATLRWEKRPYDGDR---EH-NLYVEFDMTFRF : 807
MXAN_1916 : GVNIGPEYQFYPWLTGAAGYMLGRRSSSVDGGGLNYSRHEGYARLSVTY : 421

Figure S4. Structural homology between $PgaA_{\beta b}$, GfcD, and YjbH. (A) Fold-recognition analysis of GfcD and YjbH (via HHpred) revealing N-terminal structural homology of each to the amyloid secretion β-barrel FapF (PDB: 5O65) (5) and C-terminal structural homology of each to $PgaA_{\beta b}$ (PDB: 4Y25) (2). **(B)** Multiple-sequence alignment of the $PgaA_{\beta b}$ (aa 511-807), $GfcD_{Cter\beta b}$ (aa 425-698), and $YjbH_{Cter\beta b}$ (aa 423-698) segments. Aligned residues have been coloured according to Jalview conservation score (out of 10). *Maroon*, 10; *red*, 9; *orange*, 8; *yellow*, 7; *pink*, 6. Scores of 5 or less have been omitted to improve clarity of the figure. **(C)** AlphaFold2-generated tertiary structure models for GfcD and YjbH, displayed alongside the $Pga_{\beta b}$ X-ray crystal structure for comparison. Proteins have been coloured with a spectrum, from the N-terminus (*blue*) to the C-terminus (*red*).

FIGURE S4

A)

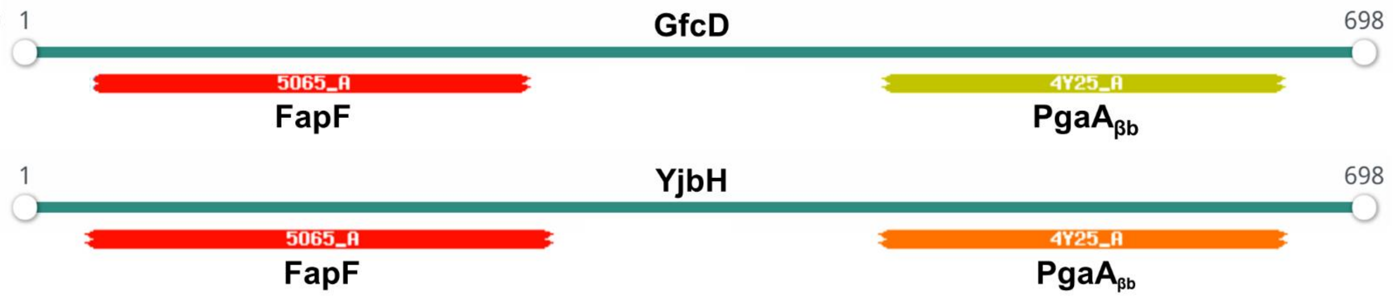

B)

```
GfcDCterβb : FSYSFNPTLSQSLGGPEDFYMFQLG----LMSSARYWFTDHLLLDGGIFTNIYNNYDKFK : 480
YjbHCterβb : FDFHIDPVLNQSVGGPENFYMYQLG----VMGTADLWLTDHLLTTGSLFANLANNYDKFN : 478
PgaAβb     : -AVDVHNLAELRIAGSTGIDAEGPDSGKHDVDLTTIVYSPPLKDNWRGFAGFGYADGQFS : 569

GfcDCterβb : SSLLPADSTLPRVRTHIRDYVRNDVYLNNLQANYFADLG--NGFYGQVYGGYLETMYAGV : 538
YjbHCterβb : YTNPPQDSHLPRVRTHVREYVQNDVYVNNLQANYFQHLG--NGFYGQVYGGYLETMFGGA : 536
PgaAβb     : EGKGIVRDWLAGVEWRSRNIWLEAEYAERVFNHEHKPGARLSGWYDFNDNWRIGSQLERL : 629

GfcDCterβb : GSELLYRPLDACWALGVDVNYVK--QRDWD---NMMRFTDYS--------TPTGFVTAYW : 585
YjbHCterβb : GAEVLYRPLDSNWAFGLDANYVK--QRDWRSAKDMMKFTDYS--------VKTGHLTAYW : 586
PgaAβb     : SHRVPLRAMKNGVTGNSAQAYVRWYQNERRKYGVSWAFTDFSDSNQRHEVSLEGQERIWS : 689

GfcDCterβb : NPPTLNGVLMKLSVGQYLAKDKG-----ATIDVAKRFDSGVAVGVWAAISNVSKDDYGEG : 640
YjbHCterβb : TPSFAQDVLVKASVGQYLAGDKG-----GTLEIAKRFDSGVVVGGYATITNVSKEEYGEG : 641
PgaAβb     : SPYLIVDFLPSLYYEQNTEHDTPYYNPIKTFDIVPAFEASHLLWRSYENSWEQIFSAGVG : 749

GfcDCterβb : GFSKGFYISIPFDLMTIGPNRNRAVVSWTPLTRDGGQMLSRKYQLYPMTAEREVPVGQ : 698
YjbHCterβb : DFTKGVYVSVPLDLFSSGPTRSRAAIGWTPLTRDGGQQLGRKFQLYDMTSDRSVNFR- : 698
PgaAβb     : ASWQKHYGTDVVTQLGYGQRISWNDVIDAGATLRWEKRPYDGDREHNLYVEFDMTFRF : 807
```

C)

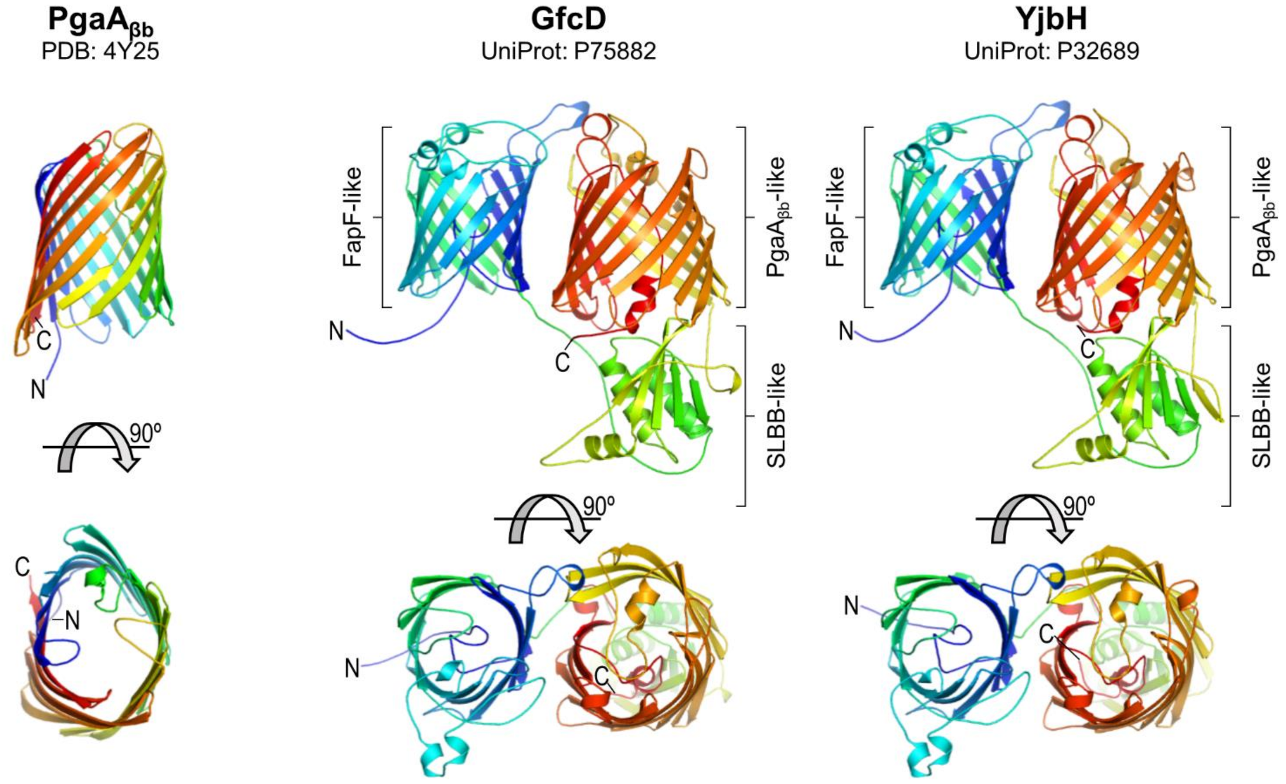

Figure S5. Panel of β-barrel query templates for synteny analysis. X-ray crystal structures and AlphaFold2 model structures of β-barrel query templates. Sequences corresponding to protein segments colored in green were used for synteny analysis. Segments colored in grey were omitted for synteny analysis. Dotted lines at the N-termini of PgaA and BcsC denote the presence of (additional) tetratricopeptide repeats in the native protein.

FIGURE S5

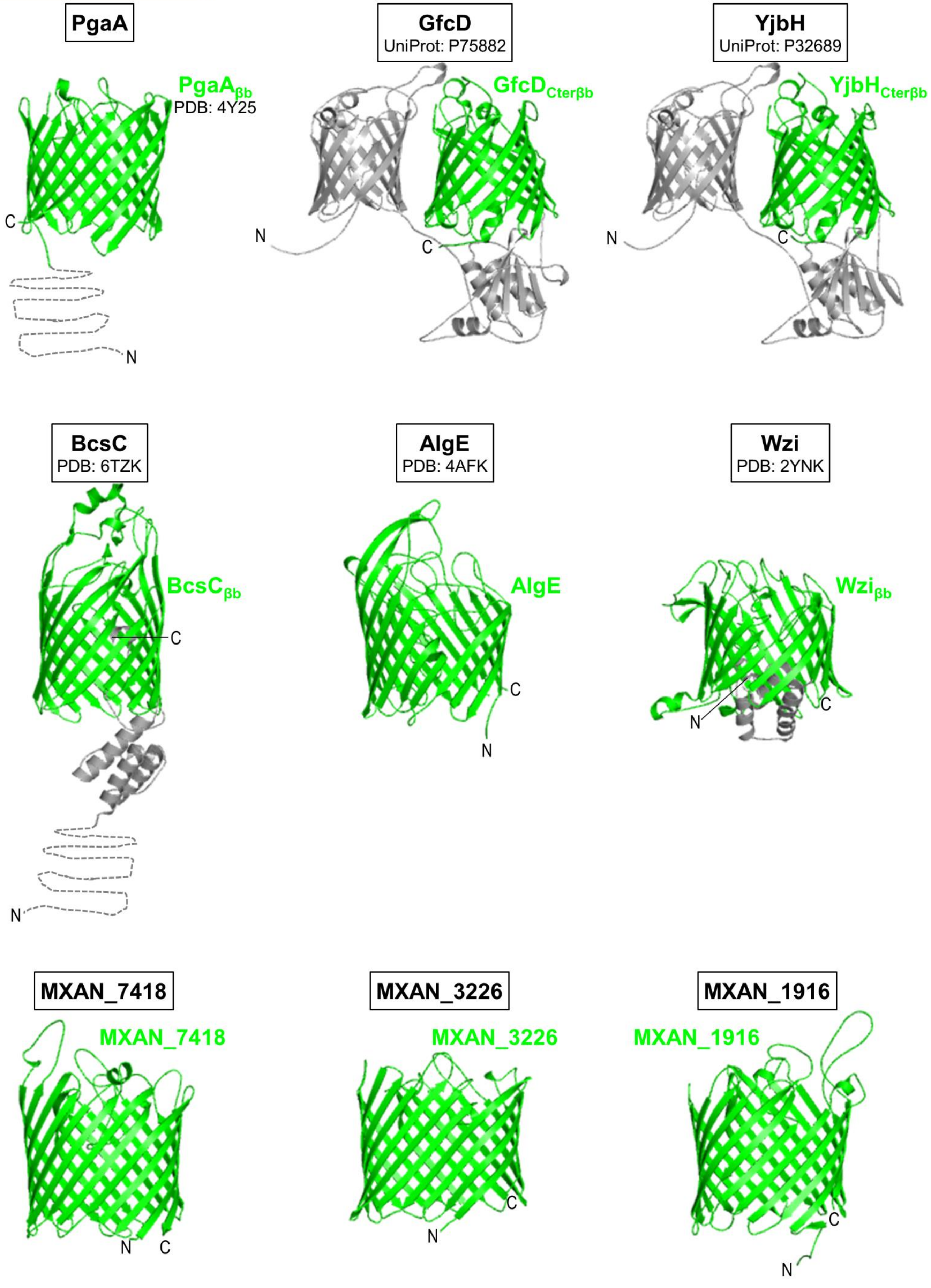
PgaA
GfcD
UniProt: P75882
YjbH
UniProt: P32689
PgaAβb
PDB: 4Y25
GfcDCterβb
YjbHCterβb
C
N
N
C
N
C
N
BcsC
PDB: 6TZK
AlgE
PDB: 4AFK
Wzi
PDB: 2YNK
BcsCβb
AlgE
Wziβb
C
C
N
N
C
N
MXAN_7418
MXAN_3226
MXAN_1916
MXAN_7418
MXAN_3226
MXAN_1916
N
C
C
N
C
N

SUPPLEMENTARY TABLE LEGENDS

Table S1. MYXO dataset analysis. (A) Protein-wise OPX classification within 61 myxobacterial genomes. **(B)** Distribution of OPX-protein types within myxobacterial genomes. **(C)** Synteny analysis of OPX-protein types with β-barrel protein homologues.

Table S2. REP dataset analysis. (A) Protein-wise OPX classification within 3662 Representative/Reference genomes. **(B)** Distribution of OPX-protein Classes within 3662 genomes. **(C)** Synteny distribution of OPX-protein types with β-barrel protein homologues. **(D)** Arrangement of syntenic OPX-protein types with β-barrel protein homologues.

Table S3. NR dataset analysis. (A) Protein-wise OPX classification within NR-database proteins.

Table S4. Distribution of OPX-protein types at Phylum, Class, Order, Family, and Genus level taxonomy.

Table S5. Evolutionary couplings between proteins. Co-evolving amino acids between PCP and OPX pairs, as well as OPX and Wzp β-barrel pairs, are presented for constituents of the EPS, BPS, and MASC pathways.

REFERENCES

1. **Mathavan I, Zirah S, Mehmood S, Choudhury HG, Goulard C, Li Y, Robinson CV, Rebuffat S, Beis K.** 2014. Structural basis for hijacking siderophore receptors by antimicrobial lasso peptides. Nat. Chem. Biol. **10:**340-342.
2. **Wang Y, Andole Pannuri A, Ni D, Zhou H, Cao X, Lu X, Romeo T, Huang Y.** 2016. Structural basis for translocation of a biofilm-supporting exopolysaccharide across the bacterial outer membrane*. J. Biol. Chem. **291:**10046-10057.
3. **Wirth C, Condemine G, Boiteux C, Bernèche S, Schirmer T, Peneff CM.** 2009. NanC crystal structure, a model for outer-membrane channels of the acidic sugar-specific KdgM porin family. J. Mol. Biol. **394:**718-731.
4. **Rutten L, Geurtsen J, Lambert W, Smolenaers JJM, Bonvin AM, de Haan A, van der Ley P, Egmond MR, Gros P, Tommassen J.** 2006. Crystal structure and catalytic mechanism of the LPS 3-*O*-deacylase PagL from *Pseudomonas aeruginosa*. Proc. Natl. Acad. Sci. U. S. A. **103:**7071-7076.
5. **Rouse SL, Hawthorne WJ, Berry J-L, Chorev DS, Ionescu SA, Lambert S, Stylianou F, Ewert W, Mackie U, Morgan RML, Otzen D, Herbst F-A, Nielsen PH, Dueholm M, Bayley H, Robinson CV, Hare S, Matthews S.** 2017. A new class of hybrid secretion system is employed in *Pseudomonas* amyloid biogenesis. Nat. Commun. **8:**263.
